# Cervical Cancer Development: Implications of HPV16 E6E7-NFX1-123 Regulated Genes

**DOI:** 10.3390/cancers13246182

**Published:** 2021-12-08

**Authors:** Kevin M. Quist, Isaiah Solorzano, Sebastian O. Wendel, Sreenivasulu Chintala, Cen Wu, Nicholas A. Wallace, Rachel A. Katzenellenbogen

**Affiliations:** 1 Herman B Wells Center for Pediatric Research, Department of Pediatrics, Indiana University School of Medicine, Indianapolis, IN 46202, USA; kmquist@iu.edu (K.M.Q.); srchinta@iu.edu (S.C.); 2 Division of Biology, Kansas State University, Manhattan, KS 66506, USA; isaiah7@k-state.edu (I.S.); sw87@k-state.edu (S.O.W.); wucen@k-state.edu (C.W.); nwallac@ksu.edu (N.A.W.)

**Keywords:** HPV16, E6, E7, NFX1-123, cervical cancer, keratinocytes

## Abstract

**Simple Summary:**

High-risk human papillomavirus (HPV) causes 4.5% of cancers and nearly all cervical cancers. HPV’s carcinogenic potential depends on its misappropriation of cellular proteins by HPV’s oncoproteins E6 and E7. High-risk HPV type 16 (HPV16) E6 binds directly to the cellular protein NFX1-123 and dysregulates proliferation, differentiation, and immunity genes. The effect of HPV16 E7 has not been studied in relation to HPV16 E6-NFX1-123-mediated dysregulation. As HPV expresses both oncogenes, and HPV carcinogenesis requires E6 and E7, it is valuable to investigate what dysregulations occur in this context. It is also important to understand their clinical and prognostic ramifications. This study’s goal was to define the gene expression profile regulated by HPV16 E6, E7, and NFX1-123 across cervical precancers and cancers, identify genes correlating with disease progression, assess patient survival, and validate findings in cell models. Finding correlates of survival and disease progression aids in biomarker identification and focuses future studies.

**Abstract:**

High-risk human papillomavirus (HR HPV) causes nearly all cervical cancers, half of which are due to HPV type 16 (HPV16). HPV16 oncoprotein E6 (16E6) binds to NFX1-123, and dysregulates gene expression, but their clinical implications are unknown. Additionally, HPV16 E7’s role has not been studied in concert with NFX1-123 and 16E6. HR HPVs express both oncogenes, and transformation requires their expression, so we sought to investigate the effect of E7 on gene expression. This study’s goal was to define gene expression profiles across cervical precancer and cancer stages, identify genes correlating with disease progression, assess patient survival, and validate findings in cell models. We analyzed NCBI GEO datasets containing transcriptomic data linked with cervical cancer stage and utilized LASSO analysis to identify cancer-driving genes. Keratinocytes expressing 16E6 and 16E7 (16E6E7) and exogenous NFX1-123 were tested for LASSO-identified gene expression. Ten out of nineteen genes correlated with disease progression, including CEBPD, NOTCH1, and KRT16, and affected survival. 16E6E7 in keratinocytes increased CEBPD, KRT16, and SLPI, and decreased NOTCH1. Exogenous NFX1-123 in 16E6E7 keratinocytes resulted in significantly increased CEBPD and NOTCH1, and reduced SLPI. This work demonstrates the clinical relevance of CEBPD, NOTCH1, KRT16, and SLPI, and shows the regulatory effects of 16E6E7 and NFX1-123.

## 1. Introduction

Human papillomavirus (HPV) is a small, non-enveloped, double-stranded DNA virus of ~8000 bp that infects mucosal and cutaneous epithelium. There are more than 200 types of HPV categorized into five different genera (pave.niaid.nih.gov (accessed on 6 December 2021)) [1]. The alpha genus is further divided into low-risk types (LR), which can cause benign epithelial lesions such as genital warts, and high-risk types (HR), which can precipitate cervical, anogenital, and head and neck cancers [2]. HPV is the most common sexually transmitted infection, and it is estimated that nearly every adult will have an infection at some point in their life [3]. Typically, the individual clears the virus [4,5,6], but in cases where infections persist, benign or oncogenic lesions can develop.

HR HPV causes 4.5% of all cancers worldwide and virtually every instance of cervical cancer, which is the fourth most common cancer in women globally [7]. Though there are effective preventive vaccines against HPV, over 300,000 women continue to die each year from cervical cancer [8]. Furthermore, HPV+ head and neck cancers are on the rise, especially in North America and northern Europe [9,10]. Of the many HR HPV types, HPV type 16 (HPV16) is responsible for the majority of all HPV+ cancers, with over 50% of cervical cancers and 70% of HPV+ head and neck squamous cell carcinomas attributable to persistent infection with this type [11,12].

The HPV16 oncogenes E6 and E7 are two of eight protein-coding genes in the viral genome; these proteins are required for cellular transformation as they prevent apoptosis and senescence through degradation of p53 and Rb [13], as well as disrupt many other regulatory pathways [14,15]. Our lab and others have shown that HPV16 E6 (16E6) binds to the host cell protein NFX1 [16,17], of which there are two isoforms: NFX1-91 and NFX1-123 [16]. The longer splice variant, NFX1-123, is highly expressed in cervical cancers and cell lines [18,19,20] and is upregulated in HPV+ compared to HPV- head and neck primary tumors [21]. It has also been shown that the protein partnership between 16E6 and NFX1-123 dysregulates genes involved in cellular growth, longevity, differentiation, and immune signaling [17,19,22,23,24,25]. A genome-wide microarray identified multiple genes upregulated by NFX1-123 in 16E6-expressing human foreskin keratinocytes (HFKs) including CEBPD, SLPI, LOR, and KRT16 [23]. Overexpression of NFX1-123 in addition to co-expression of 16E6 also led to an increase in hTERT mRNA abundance and telomerase activity [17,20,22]. Concurrently, these cells exhibited an upregulation in the expression of NOTCH1, a master cell fate regulator, as well as its canonical and non-canonical downstream targets involved in differentiation such as HES1, HES5, KRT1, and KRT10 [19,23,25]. Recently, we have shown, like NFX1-123 itself, that the expression level of many of these genes were also high in HPV16-positive primary cervical cancers relative to normal cervical tissues [18].

While the significance of the partnership between 16E6 and NFX1-123 has been demonstrated, and many of the expression changes brought about by NFX1-123 overexpression in 16E6-expressing HFKs have been observed in primary patient tumors [18,20], it is important to better understand the biological and clinical relevance of these findings. Specifically, it is valuable to understand the prognosis of cervical cancer patients whose tumors have modulated expression of these genes. When considering the typical progression of cervical cancer—from infection, to dysplasia, to different stages of cancer itself—it is critical to define whether the expression pattern of this subset of 16E6/NFX1-123-regulated genes can be predictive of disease progression. Moreover, previous studies have not investigated the role of HPV16 E7 in the context of 16E6/NFX1-123 dysregulation. As HPV infections express both oncogenes, and HPV-mediated transformation requires expression of high-risk E6 as well as E7, it is valuable to investigate what dysregulations occur as a result of high NFX1-123 expression in the context of HPV16 E6 and E7.

In the current study, we interrogated the expression of these genes in large cervical cancer clinical datasets; one containing cervical tissues with no evidence of disease, premalignant, and malignant lesions; and another containing patient outcomes linked to gene expression. This analysis helped us understand the association of 16E6/NFX1-123-regulated gene expression with disease state and survival. We found that altered expression of these genes led to improved overall, as well as disease-free, survival. Additionally, we sought to evaluate these in silico findings in an in vitro model system. Studies heretofore have focused on the interplay of NFX1-123 and 16E6, with no studies examining the role of NFX1-123 in the context of 16E6 as well as 16E7. We wanted to modify our in vitro model system to include 16E7 in order to more closely mimic a natural HPV16 infection and elucidate what regulatory changes may occur. We generated primary keratinocyte cell lines expressing 16E6 and 16E7 (16E6E7), both with overexpressed and endogenous levels of NFX1-123. These could be compared to one another, and to HPV− and HPV+ cancer cell lines. These cell lines functioned as models of HPV16 cervical dysplasia with either relatively high or basal levels of NFX1-123, and as models of HPV16 and HPV− cancers. 

## 2. Materials and Methods

### 2.1. Computational Analyses

For gene ontology, GOrilla web-based software (http://cbl-gorilla.cs.technion.ac.il/ (accessed on 15 September 2021)) was used to identify and calculate the significance of enrichments. Heatmaps with and without unsupervised clustering were generated using the heat and ggplot packages of R-software with data from GSE145976. The genes incorporated stem from a set of previously determined NFX1-123 responsive genes [23]. Least absolute shrinkage and selection operator (LASSO) and ranked stability selection was used to determine promising candidates correlating with cervical cancer disease progression from a pool of nineteen NFX1-123 responsive genes (TGM1, FBN2, HSPB1P1, PPL, SLPI, FOXA2, BNIPL, CCNB1IP1, IMPA2, RPS29, KRT16, RAET1G, LCE2B, LCE1B, ALDH3B2, NOTCH1, SPRR2G, CEBPD, LOR). The disease stage dependent gene expression data for LASSO variable selection stem from GSE145976 [26]. The LASSO computation was conducted using the GLMnet package in R-software. Training and validation were performed over 100 iterations. The output consists of a list of 10 genes that were incorporated into a model of gene expression dependent disease progression. Kaplan–Meier curves were generated using software available online at www.cbioportal.org (accessed on 11 October 2021). Statistical analysis of these curves was also completed using this software. Spearman and Pearson correlations and related statistics were calculated using the ggpubr package in R-software.

### 2.2. Plasmids and Cloning

The FLAG-tagged NFX1-123 plasmid (FN123) and LXSN control vector have been described previously [17]. pBABEpuro HPV16E6E7 was generated with Gateway Technology with Clonase II (ThermoFisher Scientific, Waltham, MA, USA, #11789020, 11791020). HPV16E6E7 was PCR amplified from an LXSN-HPV16E6E7 plasmid into which the full HPV16E6E7 sequence was previously cloned. The following attB adapter primers were used: F—GGGGACAAGTTTGTACAAAAAAGCAGGCTTTATGCACCAAAAGAGAACT; R—GGGGACCACTTTGTACAAGAAAGCTGGGTCTTATGGTTTCTGAGAACAGAT. PCR products were purified, then carried through the Gateway Clonase II protocol and cloned into a pBABEpuro gateway vector.

### 2.3. Cell Culture

Primary human foreskin keratinocytes (HFKs) were isolated from neonatal circumcision tissue by incubating in 7.5 mg/mL dispase (ThermoFisher Scientific, #17105041) for 72 h at 4 °C. The epidermis was then separated and trypsinized for 2 min before resuspending in 2% FBS in DPBS. Three biologically distinct cell lines, processed from different donor tissues, were generated (HFK1, 2, and 3) and cultured in Keratinocyte Growth Medium 2 (PromoCell, Heidelberg, Germany, #C-20011) with 1% Pen/Strep and fed every other day until the first passage. Subsequently, HFKs were cultured in EpiLife Medium with HKGS (ThermoFisher Scientific, #MEPI500CA, S0015) and 1% Pen/Strep. 293T, C33A, and SiHa cells (Gift from Dr. Galloway Lab) were cultured in DMEM (ThermoFisher Scientific, #11965092) with 10% FBS, 1 mM Sodium Pyruvate, and 1% Pen/Strep. 

### 2.4. Virus Production and Transduction

Retroviruses were produced in 293 T cells by a transient vesicular stomatitis virus G-pseudotyped production protocol that has been previously described [27]. After incubating the viral plasmids and plasmids of interest with FuGENE6 (Promega, Madison, WI, USA, #E2692) and 293 T cells, the viral supernatants were collected over the course of 48 h and stored. For transduction into HFKs, viral stocks were concentrated by ultracentrifugation, mixed with polybrene (10 μg/mL), and incubated with HFKs for three hours. After three hours incubation, the cells were washed with DPBS and refed with EpiLife medium. Then, 48 h following transduction, cells were placed under selection with either puromycin (0.5 μg/mL) or G418 (50 μg/mL). The same was done for a non-transduced control plate of HFKs. After a minimum of one week under selection as well as confirmation that non-transduced HFKs treated with puromycin or G418 were dead, typical culture medium was used. The entire population of antibiotic resistant cells were used for experimentation. In cells containing both E6E7 and either LXSN or FN123, E6E7 was transduced first and the cells underwent puromycin selection. Then, the E6E7 cells were transduced with LXSN or FN123 and underwent G418 selection. 

### 2.5. RNA Isolation, cDNA Generation, and RT-qPCR

Cells were seeded in 10 cm dishes in the following manner and harvested 48 h after plating to achieve approximately 80% confluency at time of lysis: HFKs—1–1.2 million cells; C33A—2 million cells; SiHa—1 million cells. Cells were lysed in TRIzol Reagent (ThermoFisher Scientific, #15596026) and RNA was purified according to manufacturer instructions. Then, 2 µg of total RNA was converted to cDNA using SuperScript IV VILO Master Mix with ezDNase Enzyme following manufacturer instructions (ThermoFisher Scientific, #11766050). cDNA was diluted to achieve a working concentration and quantitative real-time RT PCR was performed using the QuantStudio 3 Real-Time PCR System (ThermoFisher Scientific, #A28131). PowerUp SYBR Green Master Mix (ThermoFisher Scientific, #A25742) was used for the following primer assays: NFX1-123, FLAG, NOTCH1, KRT16, and 36B4. TaqMan Fast Advanced Master Mix (ThermoFisher Scientific, #4444557) was used for the following probe assays: CEBPD, SLPI, RAB7B, RPS29, PPL, LCE1B, FBN2, SPRR2G, and GAPDH. More details on primers and probes are included in Supplemental Table S1. Primer assays were normalized to 36B4 and probe assays were normalized to GAPDH. Each sample was assayed in technical triplicates and relative standard curves were generated for each target. Cancer cell lines were experimentally replicated three times. As each HFK cell line was isolated from a biologically distinct donor, these functioned as biological replicates. Statistical significance was calculated using an unpaired, parametric, two-tailed *t* test in GraphPad Prism. Results were considered significant if *p* value < 0.05.

### 2.6. Immunoblotting

Protein was isolated from cells seeded in the same manner as for RNA isolation as described above. RIPA buffer (150 mM NaCl, 50 mM Tris HCl pH 7.5, 0.1% SDS, 0.2% Sodium Deoxycholate, 1% NP40) with cOmplete Mini Protease Inhibitor Cocktail (Roche, #11836153001) and Phosphatase Inhibitors Cocktail 2 and 3 (MilliporeSigma, Burlington, MA, USA, #P5726, #P0044) was used for lysis. Lysates were quantified with the Pierce BCA Protein Assay Kit (ThermoFisher Scientific, #23225). Then, 35 µg of whole cell extract of each extract was separated by gel electrophoresis in 4–15%, 4–20% Mini-PROTEAN TGX gels or 16.5% Tris-Tricine TGX gels and transferred to 0.2 μM PVDF membranes using the Trans-Blot Turbo Transfer System (BioRad, Hercules, CA, USA, #4561084, 4561094, 4563063, 1704150). Primary antibodies used for protein detection and their dilutions are listed in Supplemental Table S1. Blots were visualized with SuperSignal West Pico PLUS Chemiluminescent Substrate (ThermoFisher Scientific, #34580) on the ChemiDoc Imaging System. Densitometry was calculated in ImageJ by measuring integrated density and normalizing to GAPDH. Representative blots are shown with densitometry calculated from the shown blot. Uncropped immunoblot images are shown in Supplemental Data S1.

## 3. Results

### 3.1. NFX1 Expression in Cervical Cancer Correlates with Expression of Genes Involved in RNA Binding

**Figure 1:**
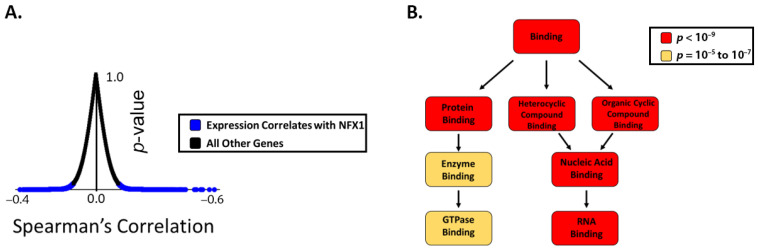
The expression of genes involved in RNA binding correlates with NFX1 expression in cervical cancer. (**A**) Plot showing the distribution of genes in the TCGA database graphed based on the extent to which their expression correlates with NFX1 on the *x*-axis (Spearman correlation coefficient) and the significance of this correlation (*p*-value). Those with a *p*-value less than 0.05 are shown in blue and considered genes with expression that correlates with NFX1 expression. All other genes are represented as black dots. (**B**) A gene ontology analysis was performed on the genes that significantly correlated with NFX1 expression (using GOrilla software). The output of this analysis is shown. Gene ontology terms are shown in boxes. The color of the box denotes the statistical significance of the enrichment, with darker colors represented greater significance. Arrows indicate increasing specificity of the gene ontology terms.

Previous publications have demonstrated that elevated expression of NFX1 correlated with changes in the expression of other genes, especially in the context of cells that are expressing 16E6 [23,25]. To identify genes whose expression correlated with NFX1 in cervical cancers, we ranked genes based on the extent to which their expression significantly correlated with NFX1 expression in the Cancer Genome Atlas (TCGA) cervical cancer data set (Supplemental Table S2). This ranking was based on their q-values derived from Benjamini–Hochberg false discover rate correction procedure. There were 13.2% (2656 of 20069) of the genes in the TCGA dataset that had a q-value less than 0.05, indicating statistically significant correlation with NFX1 expression (Figure 1A). We then used the Gene Ontology enRIchment anaLysis and visuaLizAtion (GOrilla) tool to identify enrichments among the functions of these genes. Gene ontology analysis by GOrilla identified a significant enrichment in genes involved in protein binding and RNA binding (Figure 1B). These data are consistent with known functions of NFX1 [14,17,21]. These data support that, in the context of cervical cancer, NFX1 expression correlates with other genes and pathways involved in RNA biology and, by extension, may directly or indirectly regulate RNA expression and protein amounts of genes important in cervical cancer.

### 3.2. NFX1-Regulated Gene Expression Is Altered in Dysplastic and Malignant Cervical Tissues

To determine the expression pattern of NFX1-123-regulated genes across cervical cancer development, we analyzed a previously described dataset [23] by merging transcriptomic analyses from five datasets in the National Center for Biotechnology Information Gene Expression Omnibus or NCBI GEO (GSE145976) that contained transcriptomic data linked with disease progression information [26]. This combined dataset has transcriptomic data for 262 cervical tissues including: tissue with no evidence of disease (NED, *n* = 89), premalignant lesions (CIN1 = 89, CIN2 = 22, CIN3 = 47), and cervical cancers (Stage 1 = 25, Stage 2 = 44, Stage 3 = 11). We analyzed the normalized expression of known 16E6/NFX1-123-regulated genes [18,23]. 

**Figure 2:**
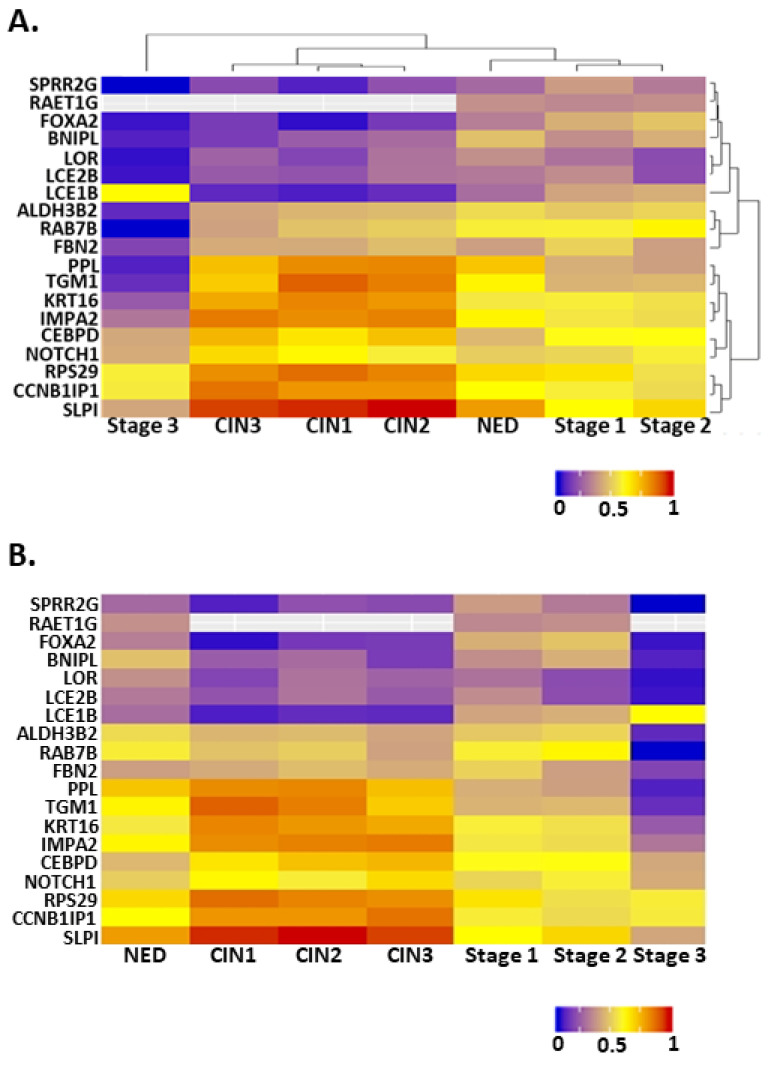
Heat maps showing the expression of NFX1-123-responsive genes at different stages of cervical cancer development. (**A**) A heat map organized by two unsupervised cluster analyses of the data. Dendrograms on the top and right show the results of these analyses. (**B**) A heat map organized along the *x*-axis such that the least transformed tissues are on the left and the most transformed are on the right. The *y*-axis lists genes in the same order as in (**A**). For all, warmer colors represent higher expression, while cooler colors represent lower expression. NED = no evidence of disease, CIN1 = cervical intraepithelial neoplasia grade 1, CIN2 = cervical intraepithelial neoplasia grade 2, and CIN3 = cervical intraepithelial neoplasia grade 3, Stage 1 = stage 1 cervical cancer, Stage 2 = stage 2 cervical cancer, and Stage 3 = stage 3 cervical cancer.

Unsupervised cluster analysis was applied to these genes with respect to disease state and gene expression. For disease state, there were three distinct clusters (Figure 2A, disease state clustering, top). Premalignant lesions formed one cluster. Cervical tissue with NED, Stage 1, and Stage 2 cancer formed a second cluster. Stage 3 cervical cancer formed its own cluster, distinct from the other two. Thus, many of the substantial changes in gene expression were seen between NED and premalignant tissues as well as different stages of cervical cancer. Unsupervised clustering by gene expression found two distinct clusters among the NFX1-responsive genes (Figure 2A, gene expression clustering, right). Approximately one third (7 of 19; SPRR2G, RAET1G, FOXA2, BNIPL, LOR, LCE2B, and LCE1B) had expression that was highest among the NED/Stage1/Stage2 cluster, lower in the premalignant cluster, and even lower in Stage 3 cervical cancer tissue. The larger cluster (9 of 19; PPL, TGM1, KRT16, IMPA2, CEBPD, NOTCH1, RPS29, CCNB1IP1, SLPI) had the highest expression in premalignant tissue and lowest in Stage 3 cervical cancer tissue. A final smaller group of three genes (ALDH3B2, RAB7B, and FBN2) had an expression pattern like the larger cluster, but without the notably increased expression in premalignant tissue. These analyses show the dynamic differences in 16E6/NFX1-123 co-regulated genes at different stages of cervical cancer progression (Figure 2B).

### 3.3. Least Absolute Shrinkage and Selection Operator (LASSO) Analysis Identifies NFX1-Regulated Genes That Are Prognostic Factors for Cervical Cancer

We next sought to identify the subset of NFX1-regulated genes that were most closely correlated with cervical cancer disease state using Least Absolute Shrinkage and Selection Operator (LASSO) analysis. This machine learning approach uses an iterative variable selection process to identify factors that have the strongest influence on disease state. LASSO analysis on the 16E6/NFX1-123 regulated genes discussed above identified a subset of ten genes (CEBPD, SPRR2G, RAB7B, NOTCH1, KRT16, FBN2, LCE1B, SLPI, RPS29, and PPL) as the most strongly correlated with disease state. 

**Figure 3:**
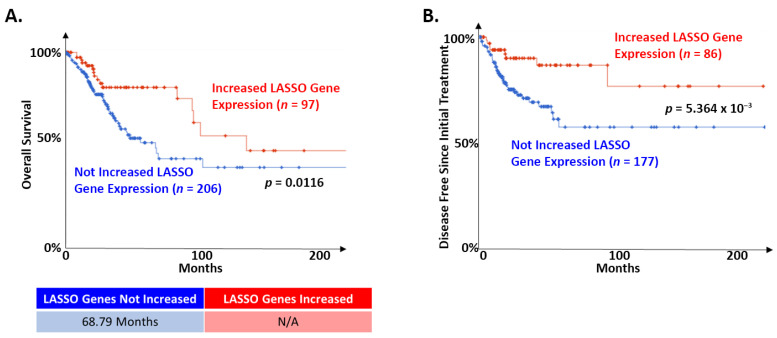
LASSO-identified genes are positive prognostic factors in cervical cancer. The TCGA cervical cancer dataset was segregated based on the expression of LASSO-identified genes as described in the text. (**A**) Of the 303 samples in the TCGA dataset with information on overall survival, 97 had altered expression of at least one LASSO-identified gene. The remaining did not have increased expression of nine LASSO-identified genes (CEBPD, SPRR2G, RAB7B, NOTCH1, KRT16, LCE1B, SLPI, RPS29, and PPL; z-score > 2). Kaplan–Meier curves of the two populations are shown. Median survival for each group is shown below the curve. N/A indicates that the group with altered expression of LASSO-identified genes did not drop below 50%. (**B**) Of the 263 samples in the TCGA dataset with information on maintenance of disease-free status after initial treatment, 86 had increased expression of at least one of the nine LASSO-identified gene analyzed in A. The remaining did not have increased expression of these genes. Kaplan–Meier curves of the two populations are shown. The median survival is not shown because neither group dropped below 50%. For all, the significance of the difference between the two groups was determined by logrank test. The relevant *p*-values are shown on each curve. Samples where LASSO-identified genes were altered are shown in red. Samples where LASSO-identified genes were NOT altered are shown in blue.

To begin assessing the potential role of these LASSO-identified genes on cervical cancer patient survival, we segregated the TCGA dataset based on the expression of each gene. Patient survival was compared, both in terms of overall survival and disease free since initial treatment survival, between cases where tumors displayed high expression (defined as having a z-score > 2 compared to control cervical tissue) and cases where they did not. There was a consistent pattern where altered expression of individual LASSO-identified genes was associated with better prognosis, but this did not reach significance. The expression of FBN2 was an exception to this pattern. High FBN2 expression was associated with significantly worse overall survival (not shown; median months survival 27.5 vs. 101.74; Logrank Test *p* = 0.0349). Based on these observations, we segregated the TCGA dataset on the presence of increased expression of 1 or more of the other nine LASSO-identified genes (z-score > 2) associated with better prognosis (CEBPD, SPRR2G, RAB7B, NOTCH1, KRT16, LCE1B, SLPI, RPS29, and PPL). In tumors with the expression of any of these genes altered in this manner, overall survival was significantly improved (Figure 3A). Further, the amount of time without disease after initial treatment was significantly improved (Figure 3B). 

### 3.4. Expression Changes of LASSO-Identified Gene in In Vitro Models of Cervical Dysplasia and Cancer

#### 3.4.1. Establishment of Cell Line Models

**Figure 4:**
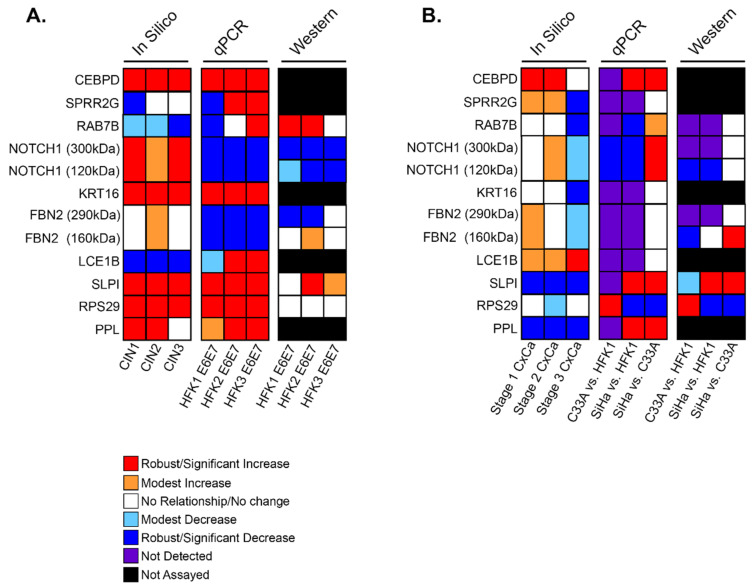
Summary of in silico and in vitro analyses of LASSO-identified genes. Expression and abundance of LASSO-identified genes/gene products is summarized. (**A**) Transcriptomic data of premalignant lesions and qPCR and immunoblot data of E6E7-expressing HFKs. (**B**) Transcriptomic data of cervical cancers and qPCR and immunoblot data in cervical cancer cells. For all, in silico data is shown with disease state increasing from left to right (i.e., The first column of in silico data is CIN1/Stage1 and the last is CIN3/Stage 3). qPCR and immunoblot data in (**A**) are shown as comparisons between parent HFK and HFK transduced with E6E7 for each biological background. qPCR and immunoblot data in (**B**) compare C33A to parent HFK1, SiHa to parent HFK1, and SiHa to C33A. For NOTCH1 and FBN2, two bands were detected by immunoblot. Information for both bands is shown and indicated in kDa sizes.

Next, it was important to quantify and compare the gene expression changes identified in our in silico analyses using in vitro cell model systems to better understand the mechanisms. To model premalignant cervical lesions, which are driven in keratinocytes during an active HR HPV infection (e.g., HPV16) by E6 and E7, HPV16E6 and HPV16E7 were stably expressed together in newly derived human foreskin keratinocytes (HFKs). This allowed us to isolate and study the effects of E6 and E7 apart from the rest of the HPV16 genome. Three 16E6E7-expressing HFK cell lines were generated from different donors for two main reasons: to allow for assessment of any variation due to biological differences between individuals, and to identify universal changes in gene expression despite this heterogeneity. To model cervical cancers, the HPV16+ cervical cancer cell line SiHa was used to represent HPV16-associated cervical cancer, and the HPV- cervical cancer cell line C33A was used to represent the rare cervical cancers that are not caused by HPV. These cell lines were validated by measuring Rb and p53 abundance via immunoblot, which showed the expected decrease in p53 and Rb in the transduced HFK cell lines (Supplemental Figure S2). Somewhat surprisingly, SiHa expressed Rb at levels equal to that of naïve HFKs, while no expression was seen in C33A. However, this antibody has been shown to clearly detect Rb in HeLa cells, so we attributed this to the properties of the antibody [28]. Furthermore, qPCR was used to detect NFX1-123 expression, which showed a significant increase in NFX1-123 transcript with the expression of 16E6E7 in HFKs and in SiHa cells (Supplemental Figure S1). PCR and immunoblot were then utilized to define RNA and protein expression of each LASSO-identified gene in these cell lines, the results of which are summarized in Figure 4A,B.

#### 3.4.2. LASSO-Identified Gene Expression in Cell Culture Models of Premalignant Lesions

Focusing first on the three 16E6E7 HFKs (HFK 1, 2, and 3) as models of cervical dysplasia with an active HR HPV infection, there were similar and reproducible expression patterns between these cell lines and in silico analyses of premalignant tissues for five of the ten LASSO-identified genes (CEBPD, KRT16, SLPI, RPS29, and PPL) (Figure 4A). RNA expression for each of these five genes was consistently elevated by qPCR as a result of 16E6E7 expression. Consistent decreases were seen for NOTCH1 and FBN2. Immunoblot analysis to quantify changes in protein levels was limited to five of the gene products to due lack of commercial antibodies. Apart from RAB7B, which was ambiguous at the transcript level across HFKs, analyses of protein amounts from immunoblots were similar to RNA.

#### 3.4.3. LASSO-Identified Gene Expression in Cell Culture Models of Cervical Cancer

Turning next to the cancer cell lines SiHa and C33A, the following comparisons were made with respect to LASSO-identified gene expression: C33A was compared to HFK1 to assess the effect of HPV-cancer dysregulation, SiHa was compared to HFK1 to examine HPV+ cancer dysregulation, and lastly SiHa compared to C33A investigated the HPV-specific effects in cancer. It was found that many LASSO-identified genes were expressed at either very low levels or not at all (Figure 4B). Overall, this pattern of expression, in these cervical cancer cell line models, was most similar to stage 3 of cervical cancers in silico, where expression levels had sharply decreased for most of the evaluated genes. SPRR2G, RA7B, KRT16, FBN2, and LCE1B RNA were either not detected or detected in very small amounts (Supplemental Figure S1, gray bars). NOTCH1 was expressed at lower levels when compared to HFK1 in both cancer cell lines. CEBPD, SLPI, and PPL, in contrast, were highly expressed in SiHa compared to HFK1. This elevated expression pattern appears to have been HPV-specific as these same genes increased relative to C33A as well. RPS29 was the only gene to be significantly increased in C33A compared to all other cell lines tested, and it was expressed at low levels in SiHa. Immunoblot results show that protein abundance followed the same trend seen at the transcript level quantified by qPCR (Supplemental Figure S2). 

#### 3.4.4. LASSO-Identified Genes of Clinical and/or Biological Interest

**Figure 5:**
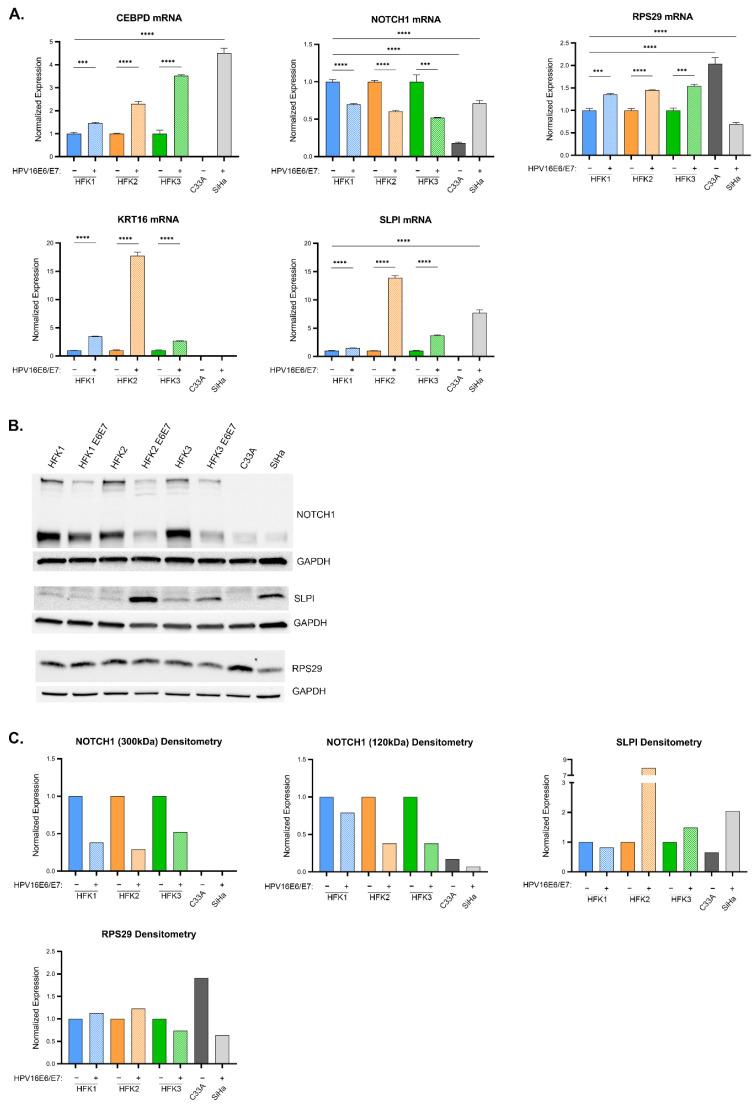
Expression of LASSO-identified genes in in vitro models of cervical disease. (**A**) CEBPD, KRT16, NOTCH1, SLPI, and RPS29 transcript levels (determined by qPCR) in HFK1-3 with and without HPV 16E6E7, as well as cancer cells C33A and SiHa. Samples are normalized to a housekeeping gene (36B4 or GAPDH) and for HFKs to their respective parent and cancer cells to HFK1. Error bars represent standard deviation for technical replicates (*n* = 3 for HFKs; *n* = 9 for cancer cells). Asterisks denote significant differences between HFK cells with and without HPV 16E6E7, and between either cancer cell or HFK1 as determined by Student *t*-Test. *p* value: *** = 0.0002; **** ≤ 0.0001. (**B**) Representative immunoblots of NOTCH1, SLPI, and RPS29 are shown. GAPDH is included as a loading control. (**C**) Densitometry of shown immunoblots (NOTCH1, SLPI, and RPS29). Samples normalized to GAPDH, and to the parent HFK for HFK1-3 and to HFK1 for the cancer cells. Two distinct bands were detected for NOTCH1 (300 kDa and 120 kDa).

**Figure 6:**
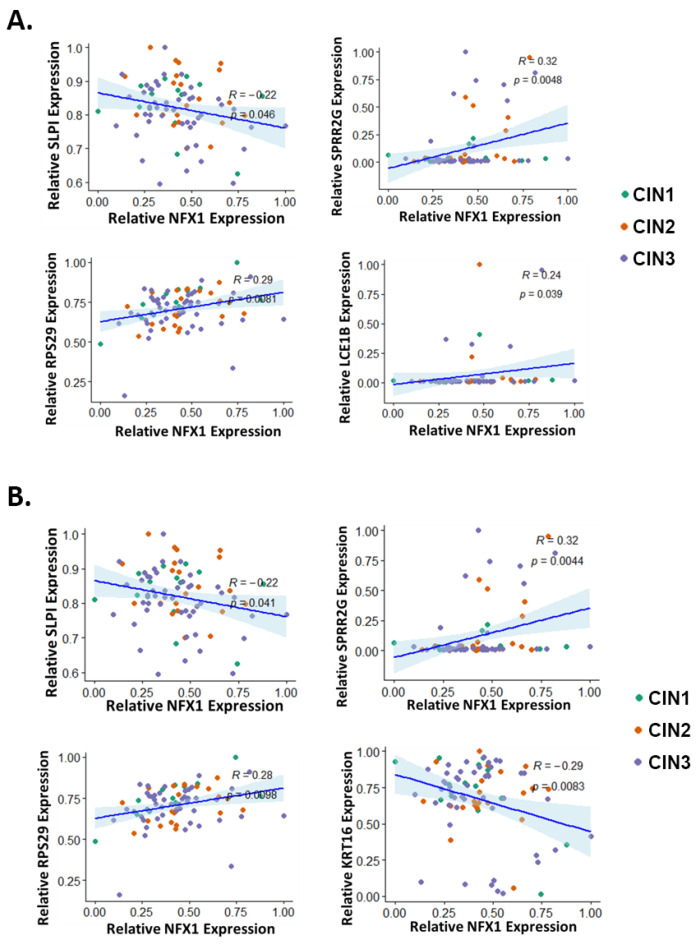
Correlation of LASSO-identified genes with NFX1 expression in premalignant cervical lesions. The expression of each LASSO-identified genes was plotted (*y*-axis) against the expression of NFX1 (*x*-axis) in premalignant cervical lesions. LASSO-identified genes whose expression has significantly correlated with NFX1 as determined by (**A**) Spearman correlation analysis or (**B**) Pearson correlation analysis is shown. For all, R and *p*-values are shown. CIN1 = cervical intraepithelial neoplasia grade 1 (green), CIN2 = cervical intraepithelial neoplasia grade 2 (orange), and CIN3 = cervical intraepithelial neoplasia grade 3 (purple).

In our dysplasia and cervical cancer models, we focused our in vitro analyses on several genes of clinical and biologic interest. CEBPD is a bZIP transcription factor with immune response functions and varied roles in cell proliferation, differentiation, and apoptosis [29]. In addition to being highly expressed in premalignant and malignant cervical cancers (Figure 2 and Figure 5), RNA levels were upregulated in HFKs with 16E6E7 expression and in the HPV16+ cervical cancer cell line, SiHa (Figure 4 and Figure 6A). The average transcriptional increase in response to 16E6E7 expression in HFKs was 2.4-fold; in SiHa cells, CEBPD expression was 4.5-fold more than in HFK1. Overall, our findings in both our dysplasia and cervical cancer cell line models corroborated in silico analyses. 

SLPI, a known immunomodulator that functions to downregulate the immune system [30], and KRT16, a stress keratin with a role in innate immunity [31], were also highly upregulated in response to 16E6E7 expression in HFKs. HFK2 E6E7 exhibited a stark increase of 10–20-fold for both of these genes; HFK1 and HFK3 E6E7 returned less pronounced, yet still statistically significant increases of 2–3-fold (Figure 5A, colored bars). SiHa did not express KRT16 but highly expressed SLPI (Figure 5A, gray bars). SLPI protein increased 8-fold in HFK2 E6E7, with more blunted changes in HFK1 and HFK3 E6E7 (Figure 5B, lanes 1–6, and Figure 5C, colored bars). RPS29 encodes a protein component of the 40 s ribosomal subunit and has an important role in translation. 16E6E7 expression in HFKs elicited a 50% average increase of RPS29 at the RNA level. Transcript levels in SiHa cells were at 70% relative to naïve HFK1, and even less compared to C33A. Densitometric analysis of immunoblotting corroborates the RNA data, with the exception of HFK3 E6E7. At the protein level, there was a modest decrease in RPS29 expression. At a transcriptional level, the expression of SLPI, KRT16, and RPS29 followed the expression seen in the in silico dysplastic cervical sample analyses. 

NOTCH1, a master cell fate regulator that has a complex role in cervical dysplasia and cancers [23] was quantified. We saw a consistent decrease in NOTCH1 mRNA of 40–50% (Figure 5A, colored bars) when 16E6 and E7 were expressed in HFKs. The antibody used for protein immunoblotting recognized two NOTCH1 isoforms: a 300 kDa band corresponding to the full-length transmembrane protein, and a 120 kDa band corresponding to the truncated protein following cleavage of the extracellular binding site by ADAM-type metalloproteases. Similar to the mRNA, there was a reduction in full-length protein of 50–75% and a reduction in the 120 kDa-sized protein of 30–75% (Figure 5B western lanes 1–6, 6C colored bars). In cervical cancer cell lines, NOTCH1 mRNA expression was increased 4-fold in SiHa cells when compared to C33A cells (Figure 5A, gray bars), although this expression remained lower than that found in HFK1. At the protein level, this reduction of NOTCH1 was even more pronounced (Figure 5B western lanes 7–8, 6C gray bars). Unlike CEBPD, SLPI, KRT16, and RPS29, NOTCH1 expression in these cell lines did not match the increase seen in cervical dysplasia and cancer samples in silico. 

### 3.5. Correlation of NFX1 Expression with the Expression of LASSO-Identified Genes

LASSO was used to identify a subset of genes, modulated by NFX1-123 in HFKs with 16E6, whose expression levels correlated with disease state in cervical tissues. However, the extent to which the expression of these LASSO-identified genes correlated with NFX1-123 in cervical tissue had not been tested. The correlation of total NFX1 (which includes both the NFX1-123 and NFX1-91 isoforms) expression with expression of each LASSO-identified gene was analyzed using Spearman and Pearson coefficients (Figure 6A and Figure 6B, respectively, and Supplemental Figures S3 and S4). In the computational analyses of NFX1-123-modulated genes, the most striking changes in expression occurred in premalignant tissue (Figure 2), hence, we chose to focus on these data. Both statistical analyses found significant correlations between the expression of three genes (SLPI, SPRR2G, and RPS29) and NFX1 expression. Both analyses also identified significant correlation between the expression of one additional gene each (Spearman: LCE1B; Pearson: KRT16) and the expression of NFX1. There was a negative correlation between expression of two genes (SLPI and KRT16) and expression of NFX1. The other three genes had a positive correlation with NFX1 expression. These data show that in premalignant cervical tissues, SLPI and KRT16 decrease with increasing NFX1 expression, and that SPRR2G, RPS29, and LCE1B increase.

The correlation data could not distinguish between NFX1 isoforms. Therefore, the effect of NFX1-123 specifically was examined in our dysplastic keratinocyte model. This was done by transducing either a FLAG-tagged NFX1-123 expression construct (FN123) or an empty vector control (LXSN) into the HFK E6E7 cell lines described above. This allowed us to focus on the effects of greater NFX1-123 expression specifically, as opposed to any and all isoforms of NFX1, in the context of 16E6E7.

**Figure 7:**
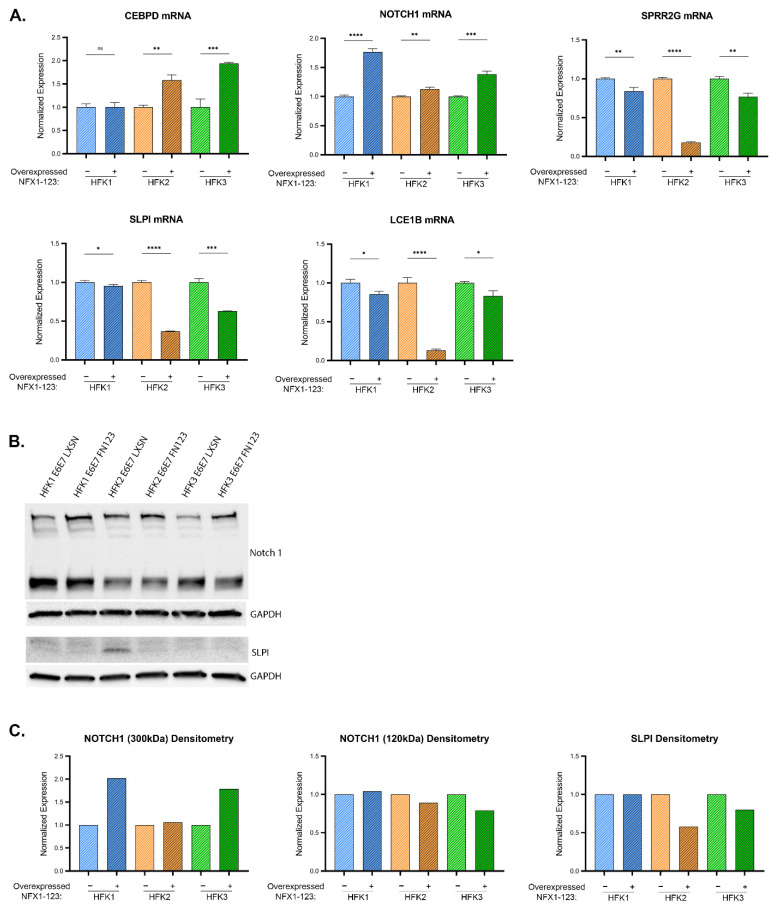
Influence of NFX1 expression on LASSO-identified gene expression. (**A**) CEBPD, SPRR2G, NOTCH1, LCE1B, and SLPI transcript levels (determined by qPCR) in HFK1-3 with and without exogenous NFX1-123 expression. Samples are normalized to a housekeeping gene (36B4 or GAPDH) and HFKs are normalized to their LXSN vector control for each pair. Error bars represent standard deviation for technical replicates (*n* = 3). Asterisks denote significant differences between HFK cells with and without exogenous NFX1-123 expression as determined by Student *t*-Test. *p* value: * = 0.03; ** = 0.002; *** = 0.0002; **** ≤ 0.0001. (**B**) Representative immunoblots of NOTCH1 (top) and SLPI (bottom) are shown. GAPDH is included as a loading control. (**C**) Densitometry of shown immunoblots (NOTCH1 and SLPI) normalized to GAPDH abundance and similarly to qPCR to each HFK LXSN vector control. Two distinct bands were detected for NOTCH1.

First, overexpression of NFX1-123 was confirmed by elevated FLAG and NFX1-123 expression in qPCR and immunoblot studies (Supplemental Figures S5 and S6). Next, the expression of LASSO-identified genes was compared between cells with endogenous expression of NFX1-123 (LXSN) and with additional, exogenous NFX1-123 expression (FN123) (Supplemental Figures S5 and S6). When reviewing the LASSO-identified genes, there was biologic variability between HFKs; however, greater expression of NFX1-123 appeared to have an additional modulatory role, beyond HPV16E6E7 itself, on five genes: CEBPD, SPRR2G, NOTCH1, LCE1B, and SLPI. CEBPD mRNA was increased between 50–100% in two of three HFK cell lines (Figure 7A). Transcript levels of SLPI decreased by an average of nearly 50%. SLPI protein expressed at low levels in all HFKs, but there was a 50% decrease in HFK2 E6E7 FN123 from its LXSN control (Figure 7A–C). NOTCH1 mRNA, which was reduced with the expression of 16E6E7 compared to naïve cells, increased by an average of 40% with exogenous NFX1-123 expression (Figure 7A). This correlated with increased full-length, 300 kDa NOTCH1 protein (Figure 7B,C). SPRR2G and LCE1B RNA levels were both reduced by an average of 40% (Figure 7A), whereas 16E6E7 expression alone did not evince a clear change in these genes across each HFK background. In sum, greater expression of NFX1-123 with co-expression of HPV 16E6E7 led to augmented NOTCH1 expression, similar to its effect seen with 16E6 alone [19,23], along with an increase in CEBPD RNA, and a decrease in SLPI, SPRR2G, and LCE1B. The increase in NOTCH1 and reduction in SLPI expression correlated with NFX1 expression in the in silico data as well (Figure 6, and Supplemental Figure S3 and S4).

## 4. Discussion

Here, we have confirmed that NFX1, based on gene ontology, functions in protein and RNA binding (Figure 1). Due to the role NFX1, and specifically the NFX1-123 isoform, plays in gene regulation at the post-transcriptional and post-translational level, we then investigated a subset of 19 genes whose expression was previously identified as correlated with or directly regulated by NFX1-123 in 16E6-expressing HFKs. Using in silico analyses of mRNA from normal, premalignant, and malignant cervical tissues, we quantified the relative expression of these genes across diagnostic categories. We found a clustering of these genes’ expression levels within pathologies (Figure 2). Furthermore, we identified a subset of 10 genes that were most predictive of cervical cancer status and survival (Figure 3). 

NFX1-123 was previously shown to cause transcriptional increases of the 19 queried genes in 16E6-expressing HFKs; however, our in silico analyses showed there is not uniform upregulation across cervical cancer progression. The unsupervised cluster analysis suggests that as persistent HPV infections lead to dysplasia and then malignancy, there is a stage-dependent organization in the way these genes are expressed (Figure 2). This is perhaps not surprising given the time it takes for HPV-associated cervical cancer to develop. The largest gene-based cluster comprising nine genes saw an increase in expression from NED to premalignancy before falling in cancer stages 1–3. Another cluster of seven saw the inverse pattern: a decrease from NED to premalignancy before returning to similar levels in the early stages of cancer. Interestingly, for nearly every gene queried, expression plummeted in stage 3 cancer. Further work is required to understand the regulatory mechanisms in place throughout cancer progression that may explain these changes. As certain viral genes become more or less active through a HR HPV infection’s life cycle, the direct consequence may be a differential expression of host genes so as to create an ideal host environment for the virus.

Alternatively, these changes may be a result of patient physiological responses to combat the infection or the developing dysplasia. This might be suggested given the Kaplan–Meier survival curve data. These data show that despite their correlation with disease progression, increased expression of LASSO-identified genes (excluding FBN2, which led to a decrease in survival) led to improved survival (Figure 3). It may be that upregulation of these genes signifies the body’s corrective response to oncogenesis rather than dysregulation directly caused by the cancer. Still another possibility might be that high expression of these genes sensitizes the cancer for treatment even while encouraging its development, something that is implied by the disease-free since initial treatment survival curve. The reason for these findings also warrants further study.

We chose to proscriptively analyze the expression of the ten LASSO-identified genes with in vitro cell culture studies of keratinocytes expressing HPV16 E6 and E7. This would allow a singular analysis of the role HPV16 E6 and E7 had in changes to gene expression, using the model of a normal keratinocyte cellular background. We also used the HPV16+ cervical cancer cell line, SiHa, and the HPV- cervical cancer cell line, C33A, to model these cancers and to parse the role of HPV16 in gene expression in those cancers. Additionally, to interrogate the role of NFX1-123 in HPV-mediated dysregulation, we overexpressed NFX1-123 by introducing exogenous FLAG-tagged NFX1-123 to the HFK cell lines expressing 16E6E7. Our study design incorporated three biologically distinct HFK cell lines (HFK1-3). This allows for underlying genetic differences to be included in our investigations of the interplay between NFX1-123, HPV16 E6, and HPV16 E7. While this likely led to variable gene expression changes in response to experimental manipulations—which can be observed, for example, in qPCR data of RAB7B following 16E6E7 transduction—we also argue that it strengthens consistent findings. A consistent alteration in gene expression—for example, SLPI mRNA following 16E6E7 transduction—across genetically distinct primary cells underscores the relevance of a given gene in the context of 16E6E7- or 16E6E7-NFX1-123-mediated dysregulation.

16E6E7 expression in HFKs elevated transcriptional expression of 5/10 LASSO-identified genes. These are CEBPD, KRT16, SLPI, RPS29, and PPL (Figure 4A and Figure 5A, and Supplemental Figure S1). NOTCH1 and FBN2 mRNA were consistently downregulated. For the majority of the 10 genes, cancer cells expressed low or undetectable levels of mRNA. HPV+ SiHa cancer cells expressed high levels of CEBPD, SLPI, and PPL mRNA relative to HFKs and HPV-C33A cancer cells. C33A expressed low or undetectable mRNA for all genes but RPS29. These results point to several possible conclusions. RPS29 mRNA may be upregulated irrespective of HPV status and because of indiscriminate cancer pathways. CEBPD, SLPI, and PPL mRNA appear to be upregulated as a result of 16E6E7 expression. KRT16 mRNA increases in 16E6E7-expressing HFKs may be dampened in more advanced cancer cells or as a result of one of the other HPV16 genes. It’s possible, too, that differences between cancer cells and HFKs are a feature of their different cellular origins.

At the protein level, 16E6E7 expression in HFKs yielded less reliable, less conclusive changes in gene expression than seen at the mRNA level. NOTCH1 decreased consistently at the protein level in response to 16E6E7 expression in HFKs, as it did at the mRNA level. SLPI, however, was somewhat difficult to detect in HFKs whereas there was robust overexpression at the mRNA level. RPS29 showed modest alterations at the protein level as well. These observations are generally true for the cancer cells as well. As the qPCR data gave more clearly quantifiable results, and because the in silico data utilized transcript data only, this work suggests the study and biomarker potential of these genes in response to HPV-mediated dysregulation is more useful at the mRNA level.

The qPCR data for 16E6E7-expressing HFKs demonstrated considerable similarity to the computational findings of mRNA expression in cervical dysplasia. Five out of ten consistently upregulated genes (CEBPD, KRT16, SLPI, RPS29, and PPL) were also increased in cervical dysplasia. This points to the apparent importance of 16E6 and or 16E7 expression for the modulation of these genes in cervical transformation. Where our cell model did not corroborate the in silico data, there may be additional features of the virus that lead to the changes seen in the clinical data. For example, the increase in NOTCH1 mRNA in cervical dysplasia may be a result of another HPV gene.

The transcriptional expression of LASSO genes exhibits stage-dependent organization in cancer stages 1–3. As stated, for nearly every gene, expression drops significantly in stage 3 cancer. LCE1B is the only transcript that increased as cancer stages advanced. CEBPD expressed at high levels during stage 1 and 2 cancer. KRT16 mRNA expressed at modest levels in cancer stages 1 and 2. SLPI and PPL exhibited a drastic shift in their expression patterns from cervical dysplasia to cancer, expressing at low levels. Further studies are needed to understand the dynamic expression patterns of these genes through cervical cancer. Ultimately, 16E6E7 HFKs better modeled cervical dysplasia than cervical cancer. SiHa cells and 16E6E7 HFKs both expressed high CEBPD, SLPI, and PPL. For other genes, SiHa cells expressed low or undetectable levels of transcript, which for genes like KRT16 or RPS29, demonstrated SiHa to be a good model. A shortcoming of the cell models is the inability to examine the effects of the surrounding physiological events that occur as cancer develops. 

CEBPD, KRT16, SLPI, RPS29, and PPL displayed significant transcriptional changes across 16E6E7-HFKs backgrounds that were consistent with premalignant tissues. CEBPD and KRT16, in particular, were two LASSO-identified genes with strong positive coefficients. CEBPD is a basic leucine zipper transcription factor with regulatory roles in many domains, and KRT16 is a structural protein in epithelial cells. Both are immune regulators that respond to inflammation. NOTCH1, though it decreased at the transcript and protein level in 16E6E7 HFKs and in cancer cells, exhibited high expression in cervical dysplasia and a positive LASSO coefficient. NOTCH1 is a critical regulator of cellular proliferation and differentiation. Aberrant regulation of this gene may involve additional viral genes, or aberrantly expressed host genes like NFX1-123. The mutual support between the in silico and in vitro studies for these genes, as well as their known functional roles point to their potential roles as biomarkers for cervical cancer development. 

The changes brought about by exogenous NFX1-123 expression in the context of 16E6E7 expression suggest additional modulatory roles of this gene within HPV+ dysregulation. It has been shown that NFX1-123 is highly expressed in cervical cancer and in HPV+ cancer cell lines, that NFX1-123 binds to 16E6, and that this partnership affects expression of proliferation and differentiation markers [17,18,19,20,23,25]. Previous data also shows that 16E6-expressing HFKs with overexpressed NFX1-123 upregulate the subset of genes [23]; the effect of 16E7, however, had not been examined in this context. Our in vitro studies show that in some instances overexpressed NFX1-123 synergizes the effect of 16E6E7 with respect to expression of LASSO-identified genes. Such is the case with the expression of CEBPD. In other cases, exogenous NFX1-123 appears to have a mitigating role to that of 16E6E7. This is seen with the expression of NOTCH1, and SLPI, where increased NFX1-123 mRNA corresponded to an increase in NOTCH1 and a decrease in SLPI. SPRR2G and LCE1B were relatively unchanged by the introduction of 16E6E7 into HFKs, but clearly decreased with overexpressed NFX1-123. The viral oncogenes may require high levels of NFX1-123 to downregulate these genes. We posit that these variations in gene expression dynamics document the evolutionary nature of HPV associated cervical cancer development and progression and the partnership cellular host factors play throughout this longitudinal process.

## 5. Conclusions

In conclusion, our findings using in silico analyses and in vitro model systems of HPV+ dysplasias and cancers uncovered gene alterations correlated with cervical cancer progression and regulated by NFX1-123 and HPV16 E6 and E7. 16E6E7-expressing HFKs with and without overexpressed NFX1-123 served as a model for cervical dysplasia. qPCR data of queried genes yielded results that corroborated transcriptional expression patterns of cervical dysplasia and LASSO analysis identified genes correlated with advancing disease stages. These genes, particularly CEBPD, SLPI, KRT16, and NOTCH1, may serve as potential biomarkers at the mRNA level for the course of cervical cancer development, and warrant further study as it relates to understanding HPV-dependent carcinogenesis.

## Data Availability

We analyzed previously published datasets, so no new datasets were produced.

## Supplementary Materials

The following are available online at https://www.mdpi.com/article/10.3390/cancers13246182/s1, Table S1: qPCR primers and probes and immunoblot antibodies, Table S2: Ranked NFX1-correlating genes in cervical cancer, Figure S1: full qPCR data of 16E6E7 HFKs and cancer cells, Figure S2: full immunoblot data of 16E6E7 HFKs and cancer cells, Figure S3: Correlation of NFX1 and LASSO genes—Spearman, Figure S4: Correlation of NFX1 and LASSO genes—Pearson, Figure S5: full qPCR data of NFX1-123 overexpression HFKs, Figure S6: full immunoblot data of NFX1-123 overexpression HFKs, Data S1: uncropped immunoblot images.
