# Supplementary material for: Cervical Cancer Development: Implications of HPV16 E6E7-NFX1-123 Regulated Genes
Source: Cancers (Basel). 2021 Dec 8;13(24):6182. doi: 10.3390/cancers13246182 (PMC8699269; doi:10.3390/cancers13246182)
Supplement: Supplementary file 1 [file cancers-13-06182-s001.zip › cancers-1474260-suppl-figures.pdf]

Supplementary figures

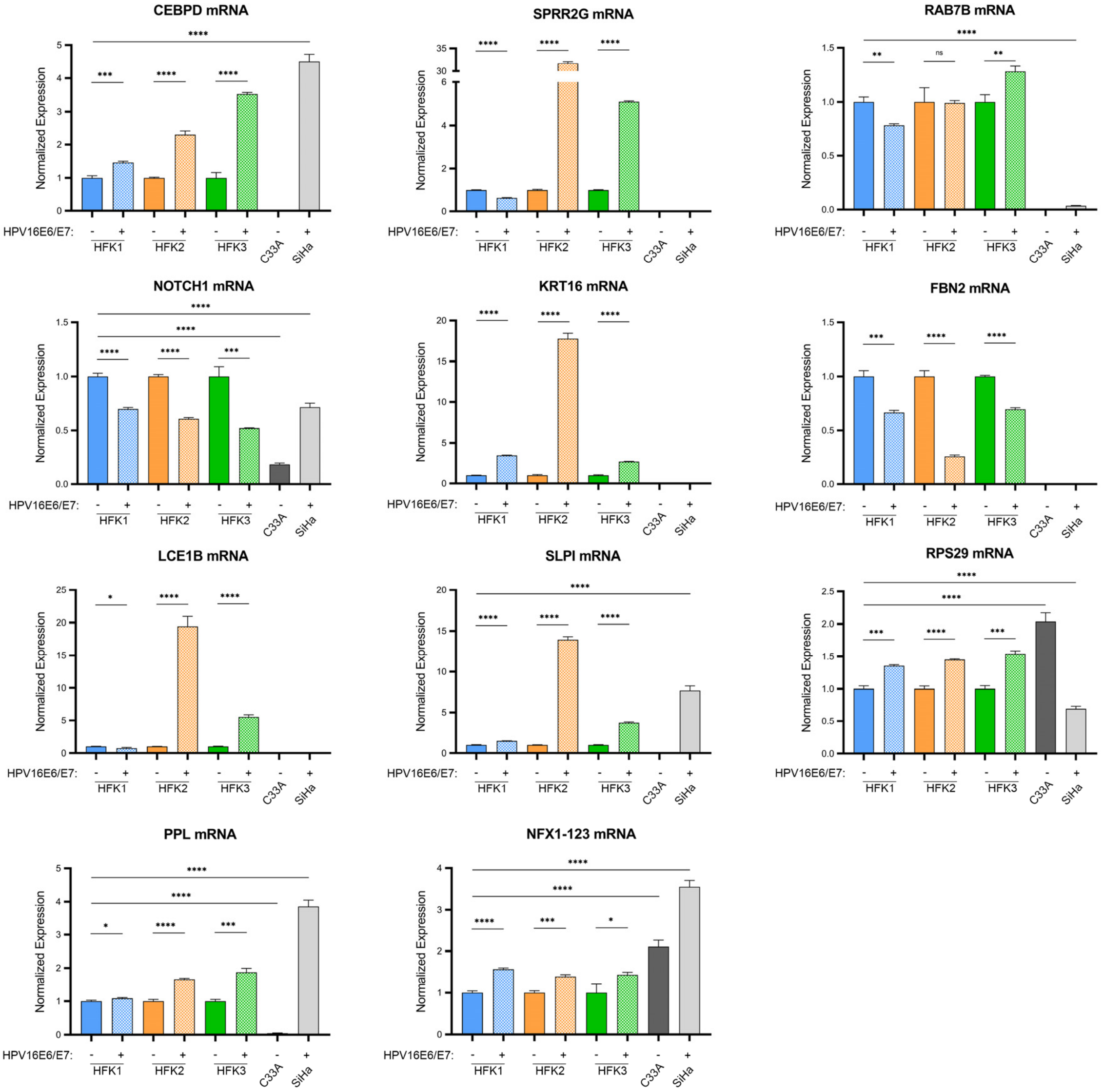

Figure S1. full qPCR data of 16E6E7 HFKs and cancer cells.

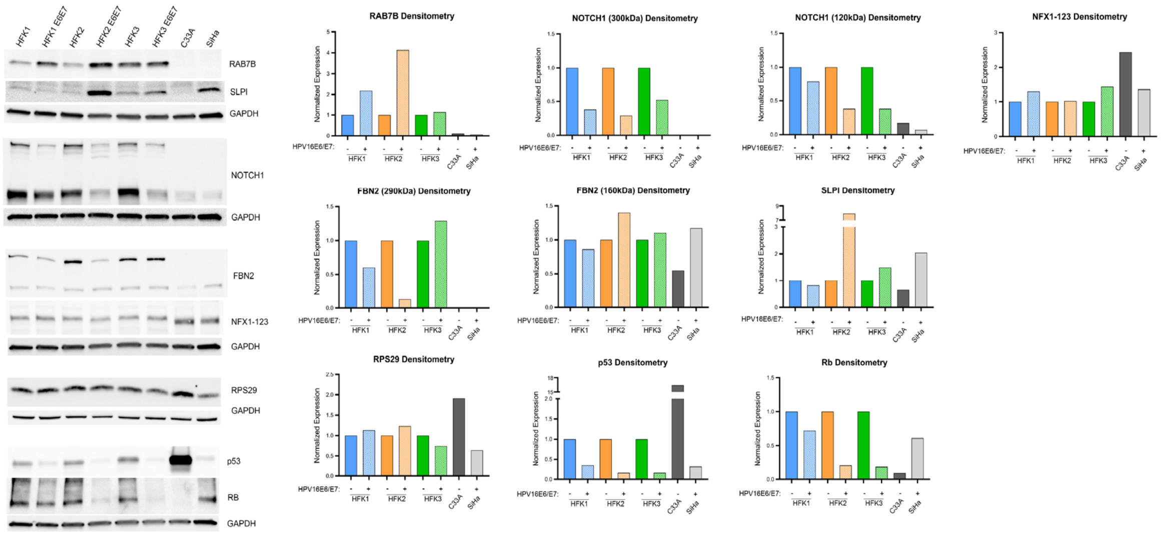

Figure S2. full immunoblot data of 16E6E7 HFKs and cancer cells.

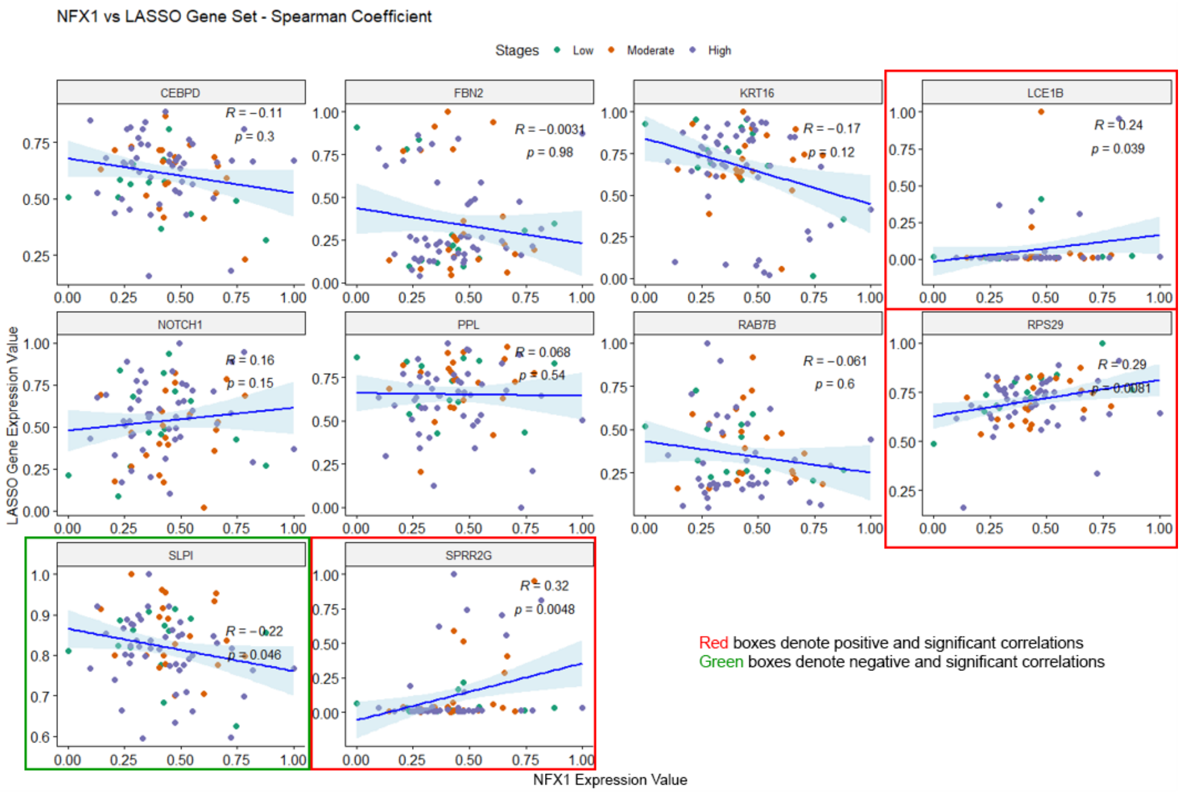

Figure S3. Correlation of NFX1 and LASSO genes—Spearman.

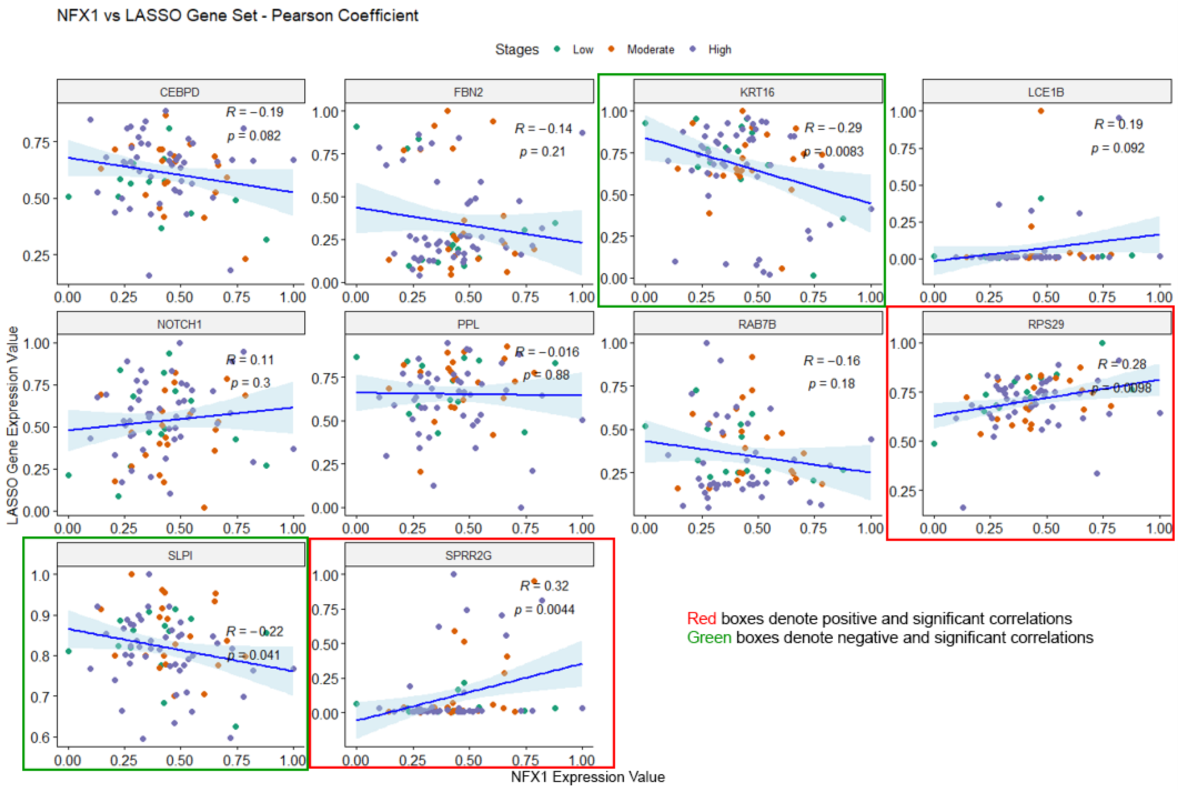

Figure S4. Correlation of NFX1 and LASSO genes—Pearson.

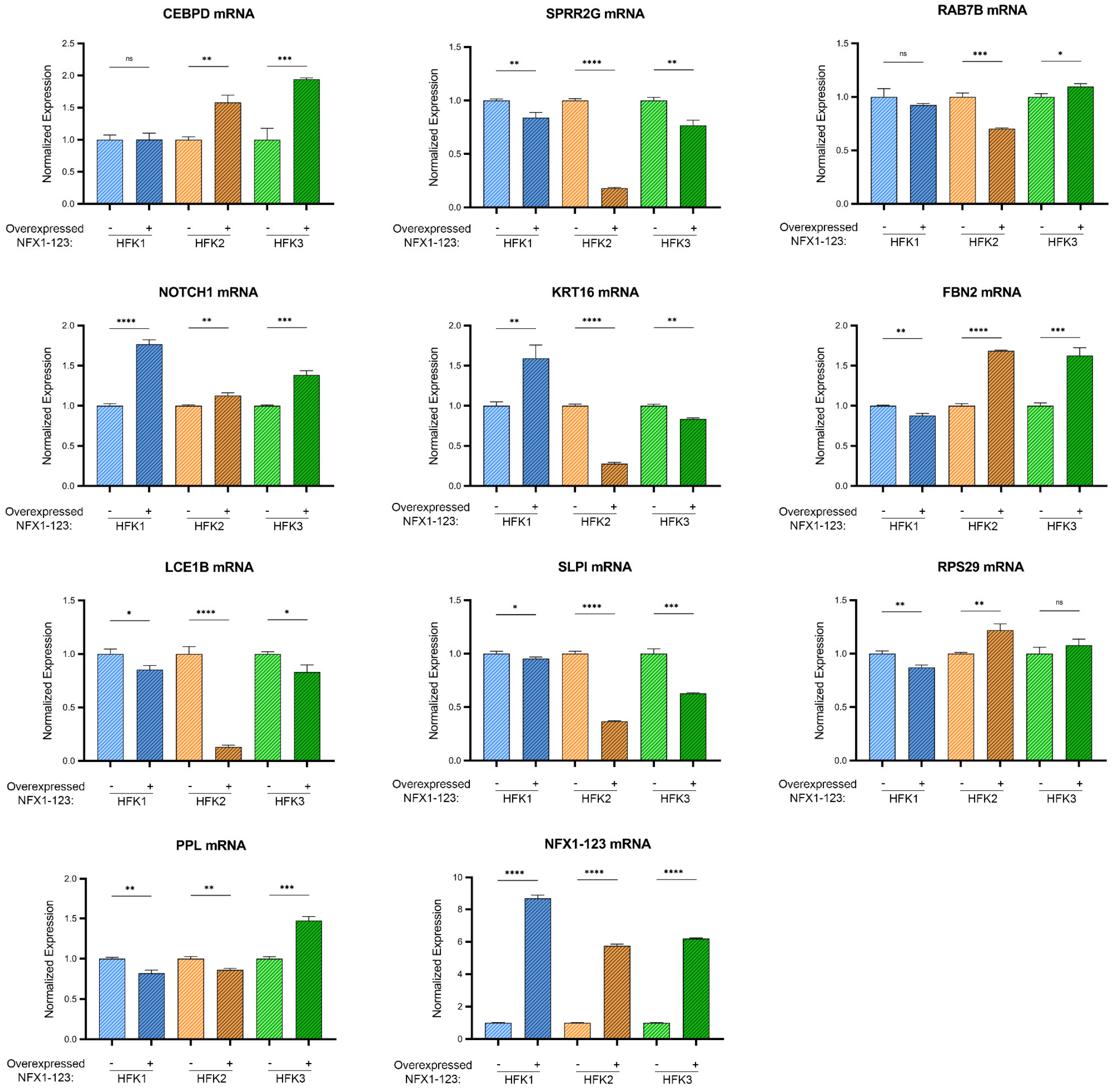

Figure S5. full qPCR data of NFX1-123 overexpression HFKs.

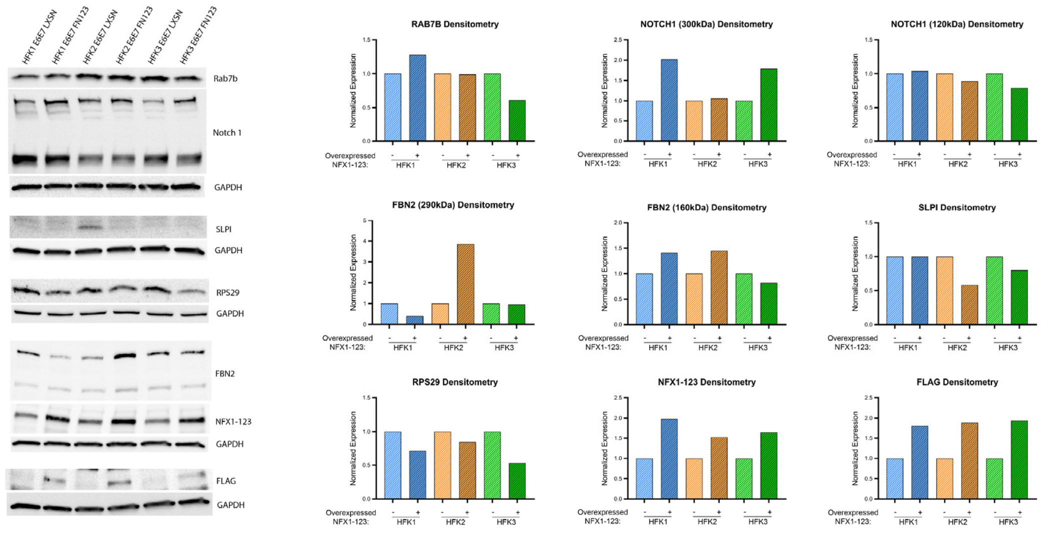

Figure S6. full immunoblot data of NFX1-123 overexpression HFKs.
