# Supplementary material for: Cervical Cancer Development: Implications of HPV16 E6E7-NFX1-123 Regulated Genes
Source: Cancers (Basel). 2021 Dec 8;13(24):6182. doi: 10.3390/cancers13246182 (PMC8699269; doi:10.3390/cancers13246182)

Full Blot Images

Antibody	Company/Cat. #	Primary Dilution	Secondary	Secondary Dilution
RAB7B	Novus Biologicals – H00338382-B01P	1:1200	Mouse-HRP	1:2500
NOTCH1	Cell Signaling – #3447	1:1000	Rat-HRP	1:2000
SLPI	R&D Systems – AF1274	1:500	Goat-HRP	1:2000
RPS29	Abcam – ab56224	1:2500	Rabbit-HRP	1:2000
FBN2	ProteinTech – 20252-1-AP	1:750	Rabbit-HRP	1:2000
NFX1-123	Homemade. Kind gift of Ann Roman.	1:1200	Rabbit-HRP	1:2000
FLAG	Millipore-Sigma – F3165	1:1000	Mouse-HRP	1:2000
GAPDH	Abcam – ab8245	1:125,000	Mouse-HRP	1:2500
p53	Santa Cruz – sc-126	1:1500	Mouse-HRP	1:2500
Rb	Cell Signaling – #9309	1:1500	Mouse-HRP	1:2500

All blots blocked and antibodies incubated in 4% non-fat dry milk in TBS-T

Blot 1
FLAG 1:1000
Mouse-HRP 1:2000

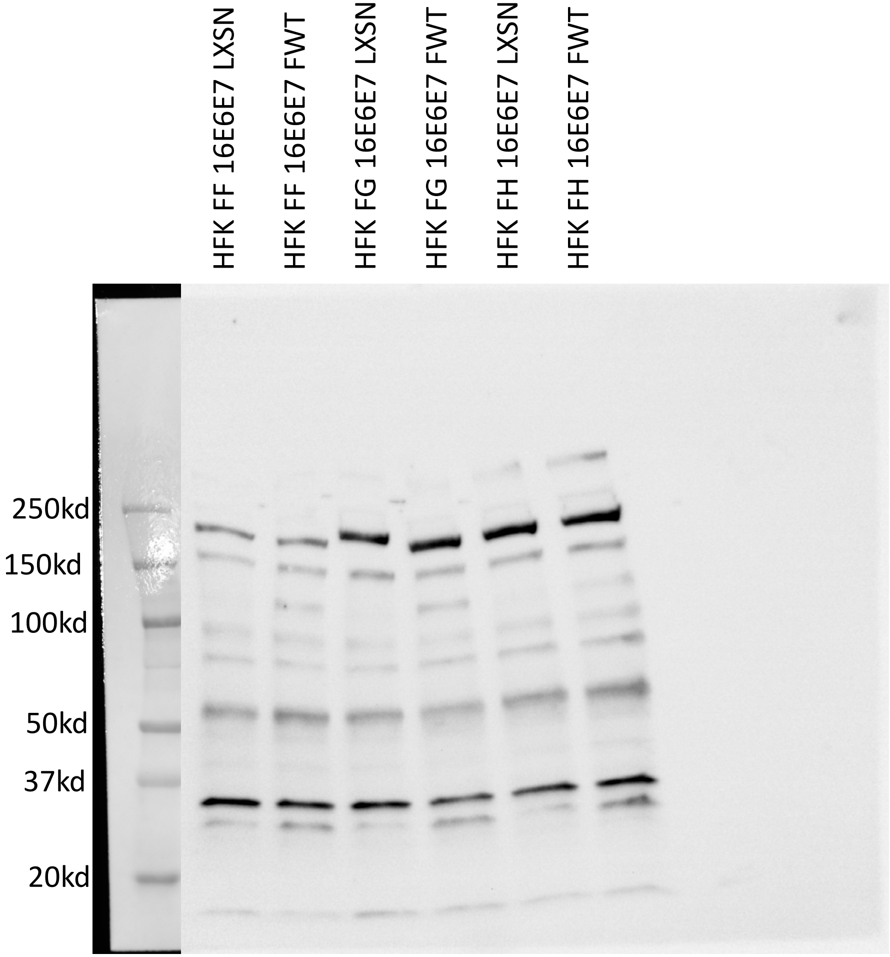

Blot 1
GAPDH 1:125,000
Mouse-HRP 1:2500

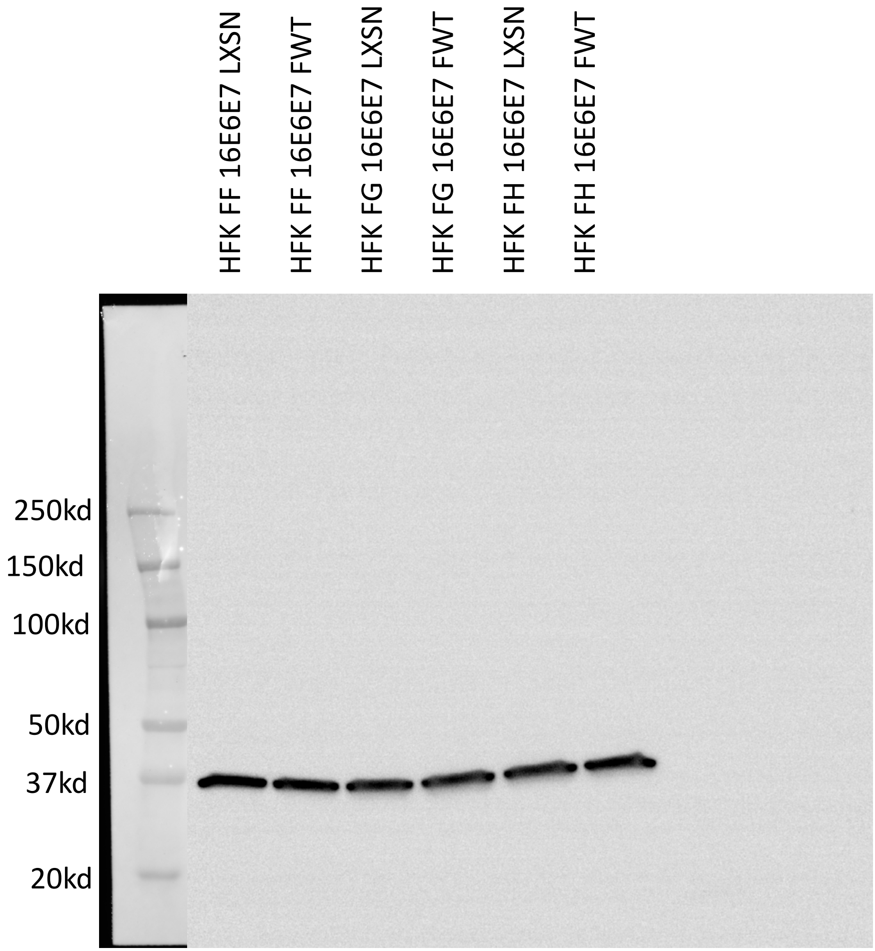

Blot 2
Notch 1 1:1000
Rat-HRP 1:2000

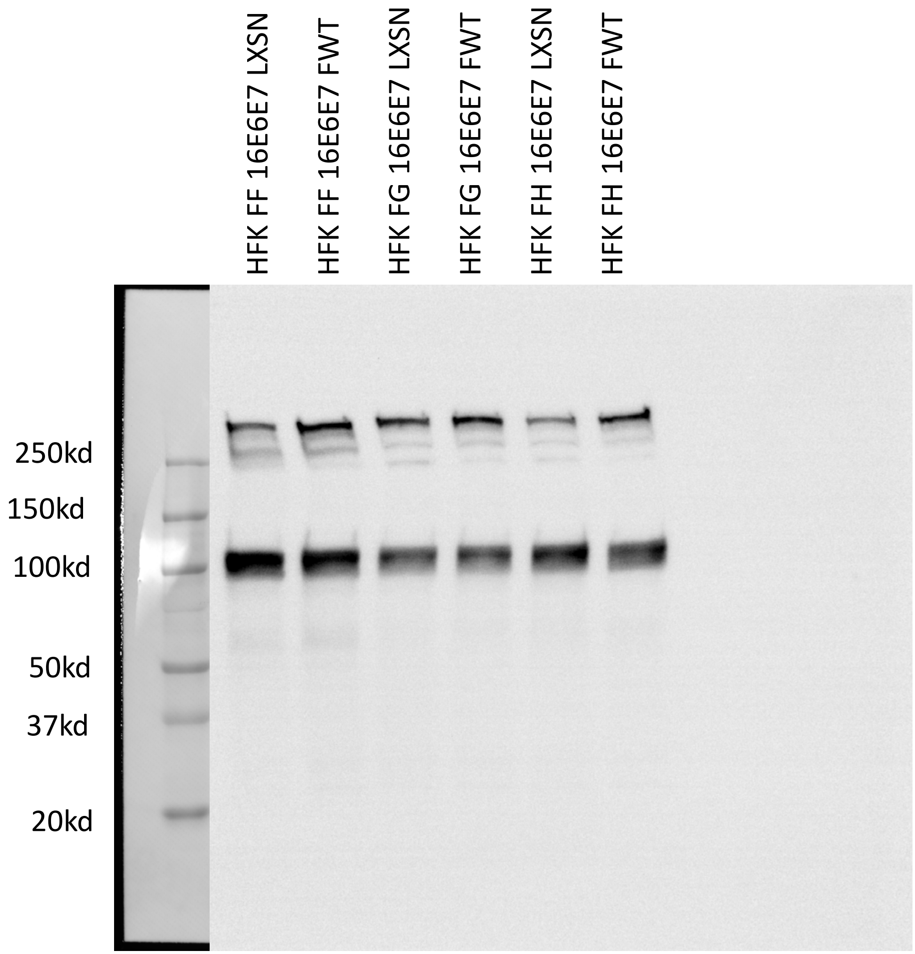

Blot 2
RAB7B 1:1200
Mouse-HRP 1:2500

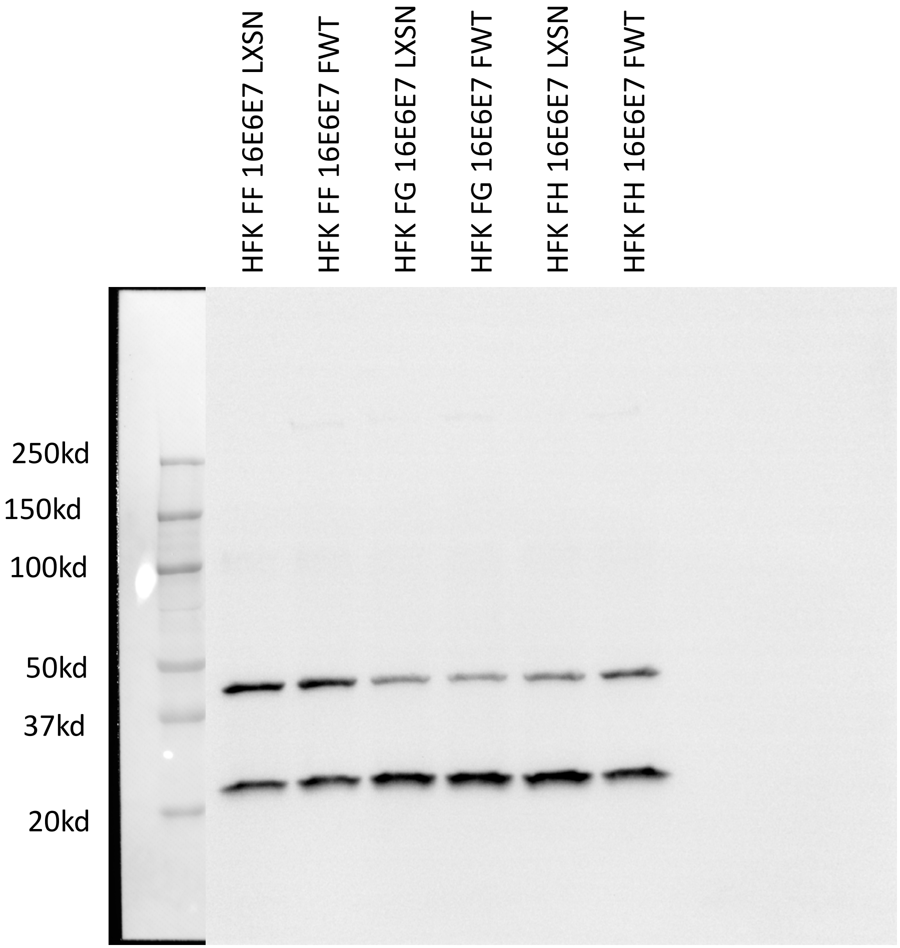

Blot 2
GAPDH 1:125,000
Mouse-HRP 1:2500

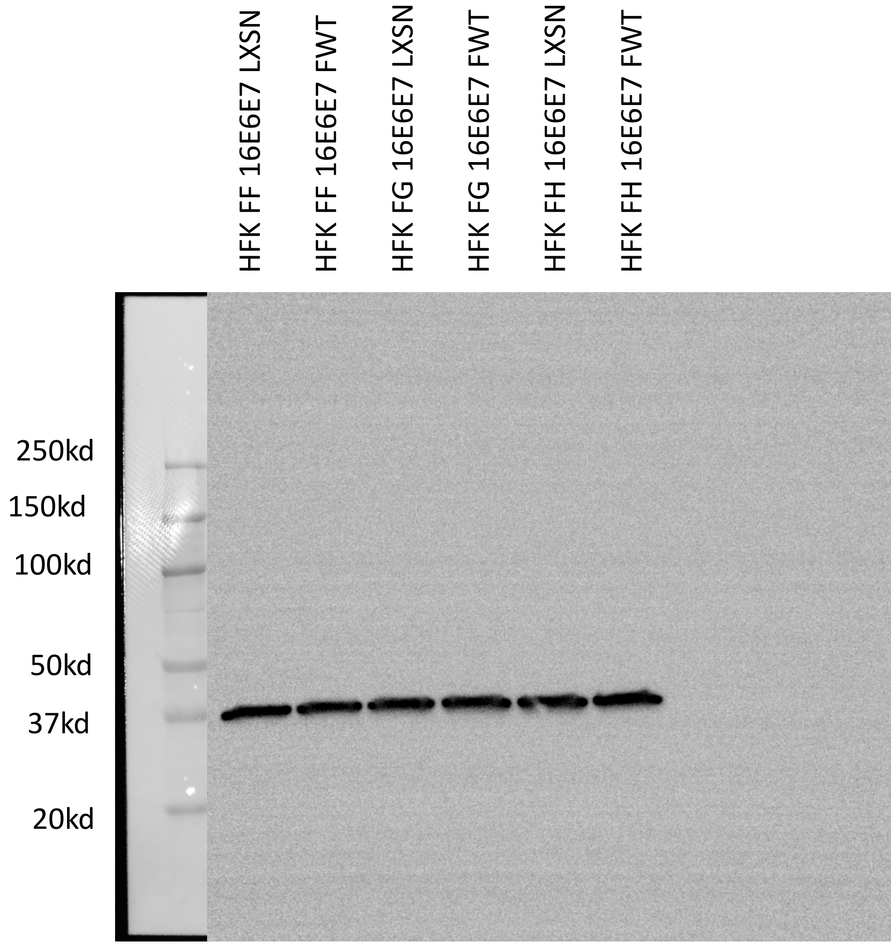

Blot 1
Fbn2 1:750
Rabbit-HRP 1:2000

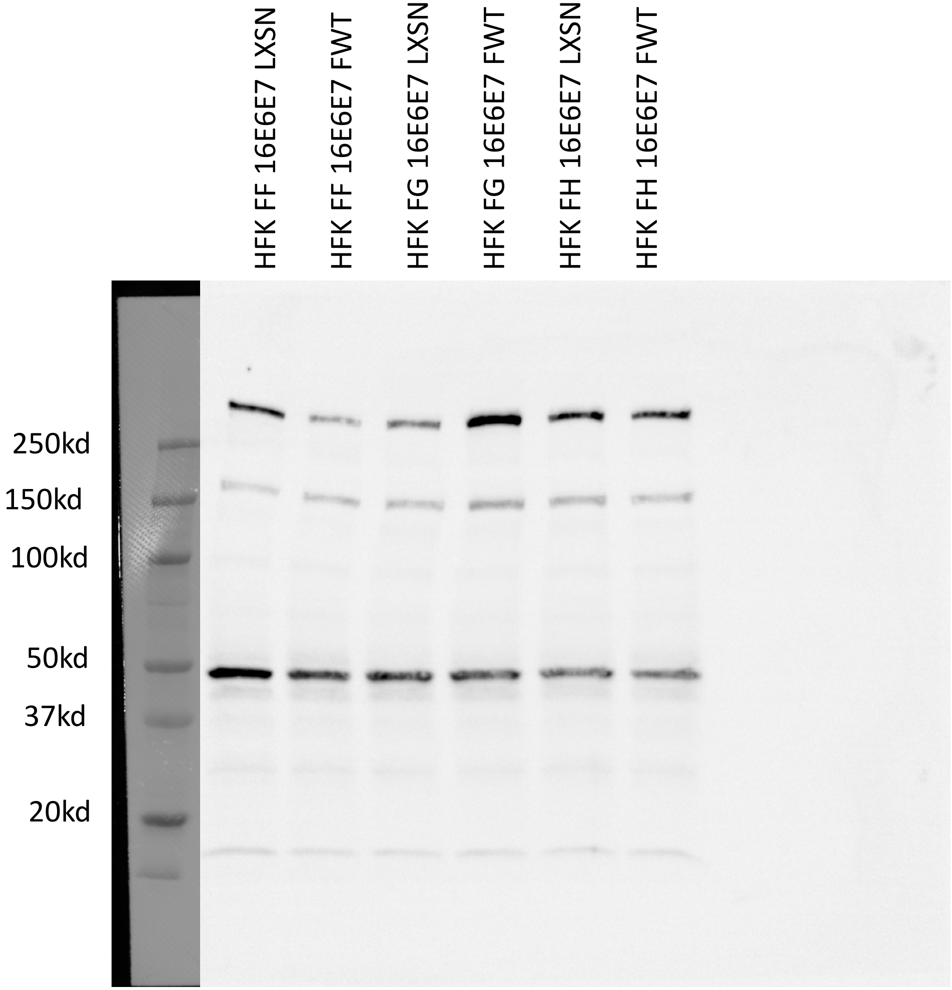

Blot 1
Nfx1-123 1:1200
Rabbit-HRP 1:2000

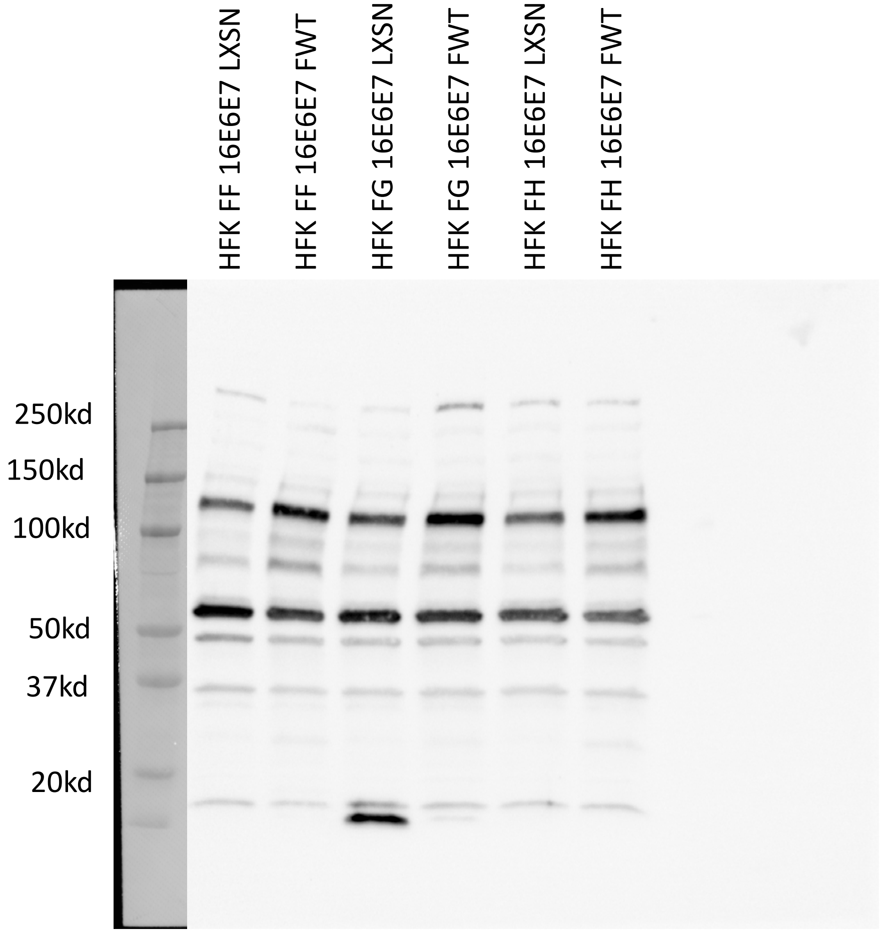

Blot 1
GAPDH 1:125,000
Mouse-HRP 1:2500

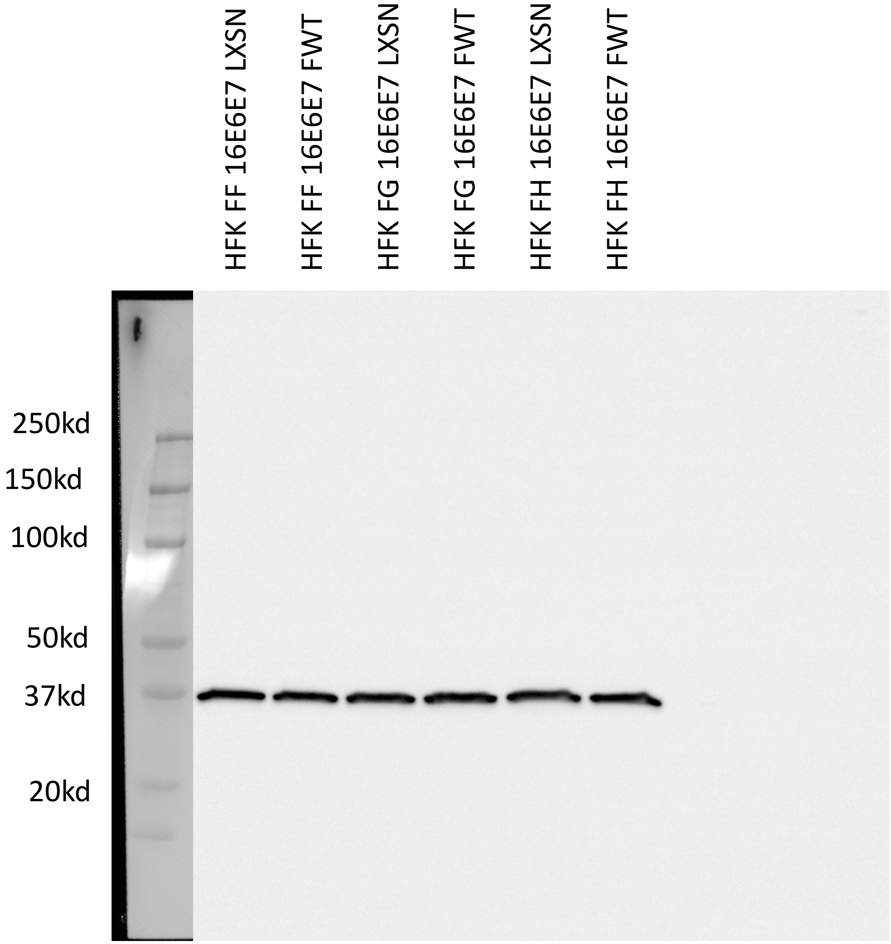

SLPI 1:500 Goat-
HRP 1:2000

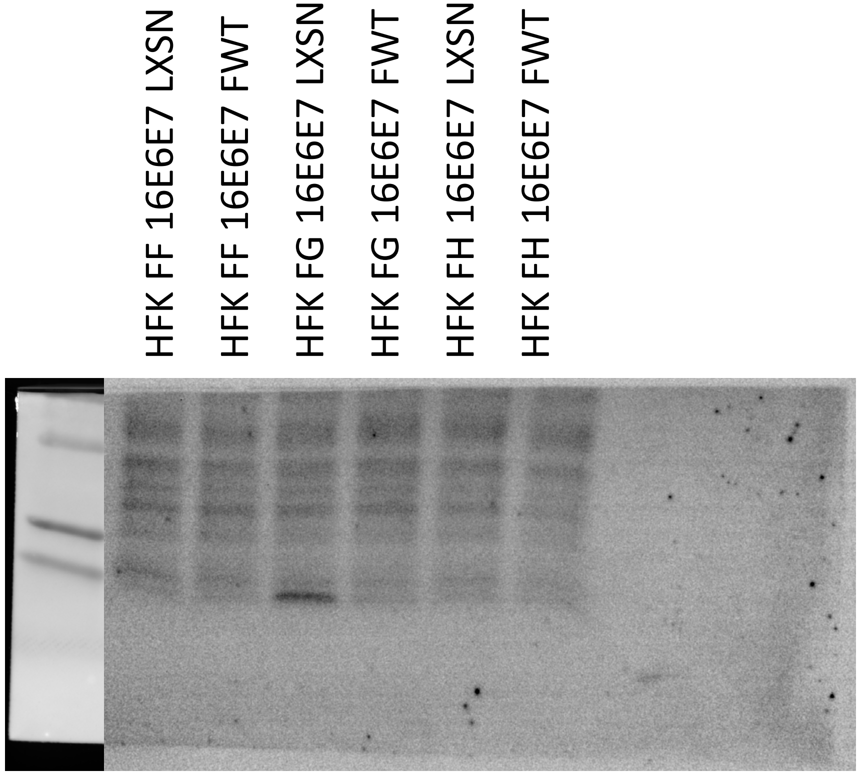

GAPDH 1:125,000
Mouse-HRP 1:2500

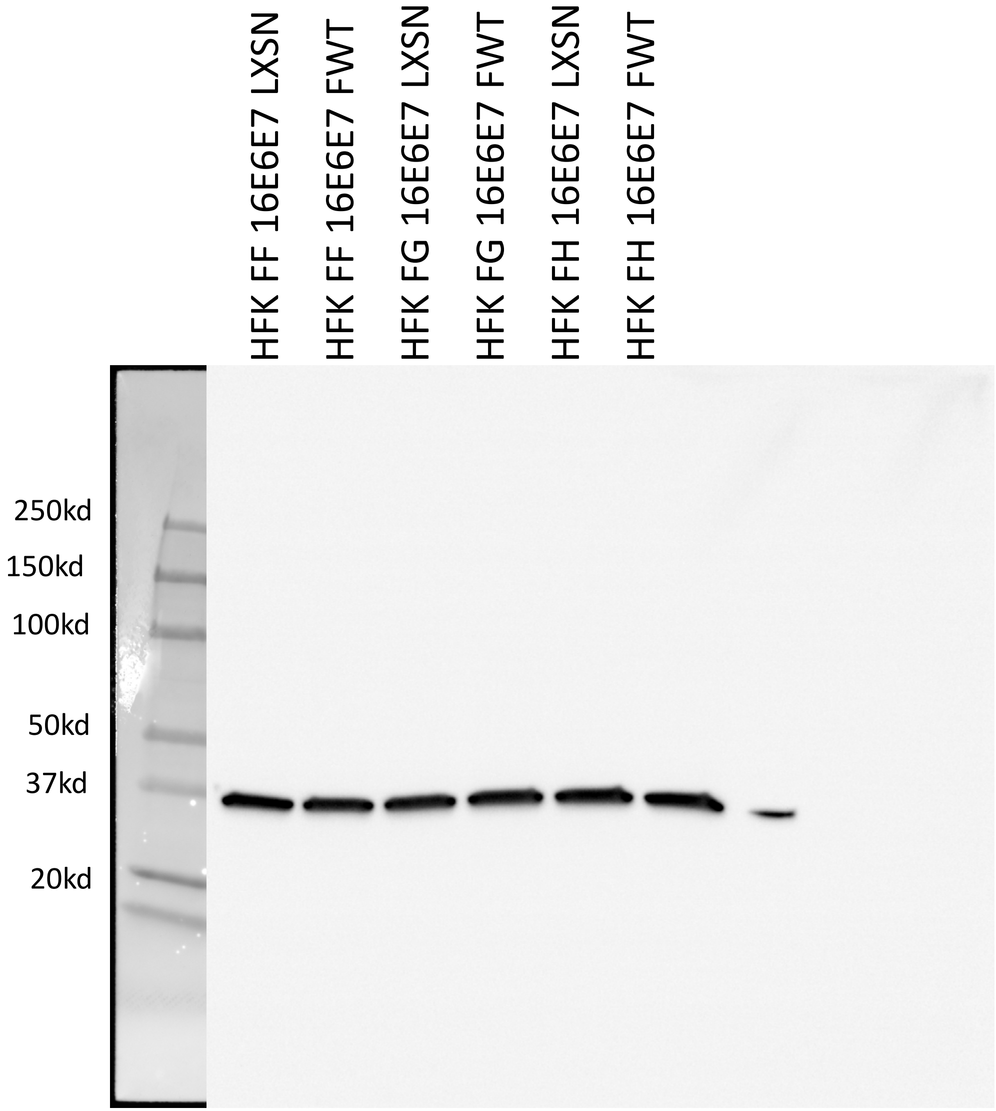

Blot 2
RPS29 1:2500
Rabbit-HRP 1:2000

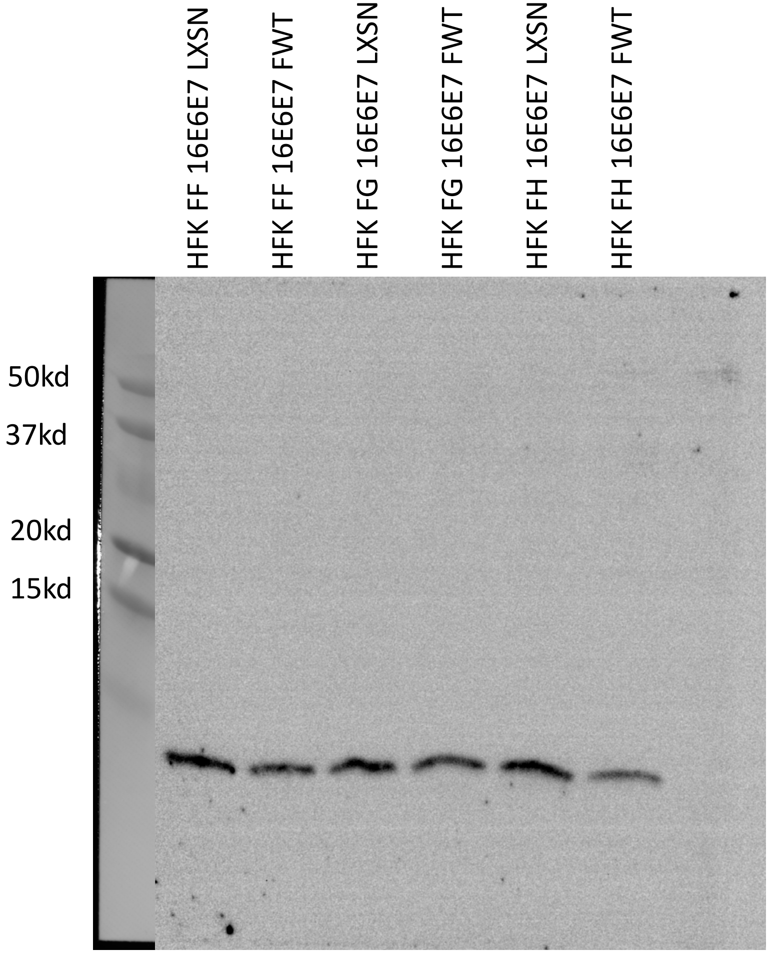

Blot 2
GAPDH 1:125,000
Mouse-HRP 1:2500

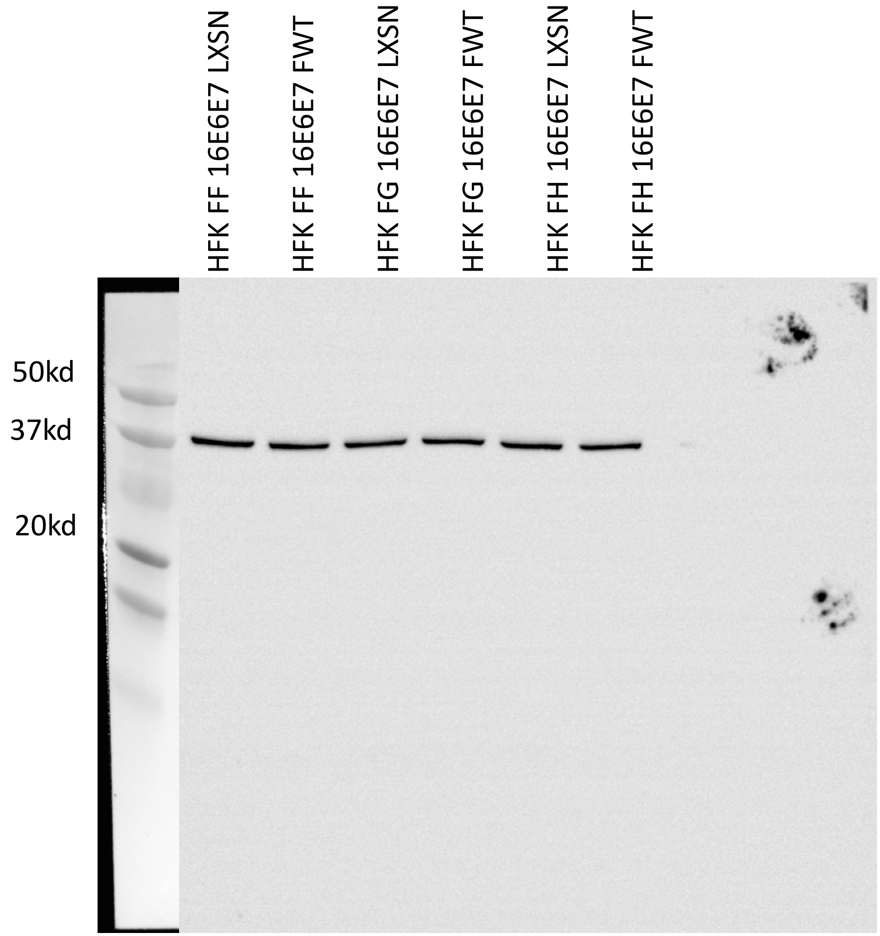

Blot 1
SLPI 1:750 Goat-
HRP 1:2500

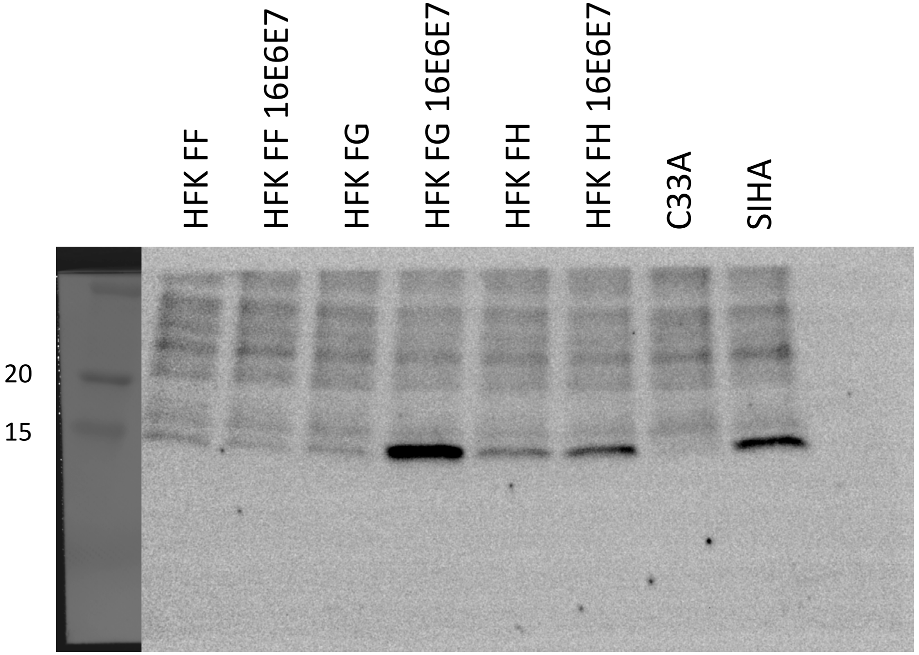

Blot 1
RAB7B 1:1000
Mouse-HRP 1:2500

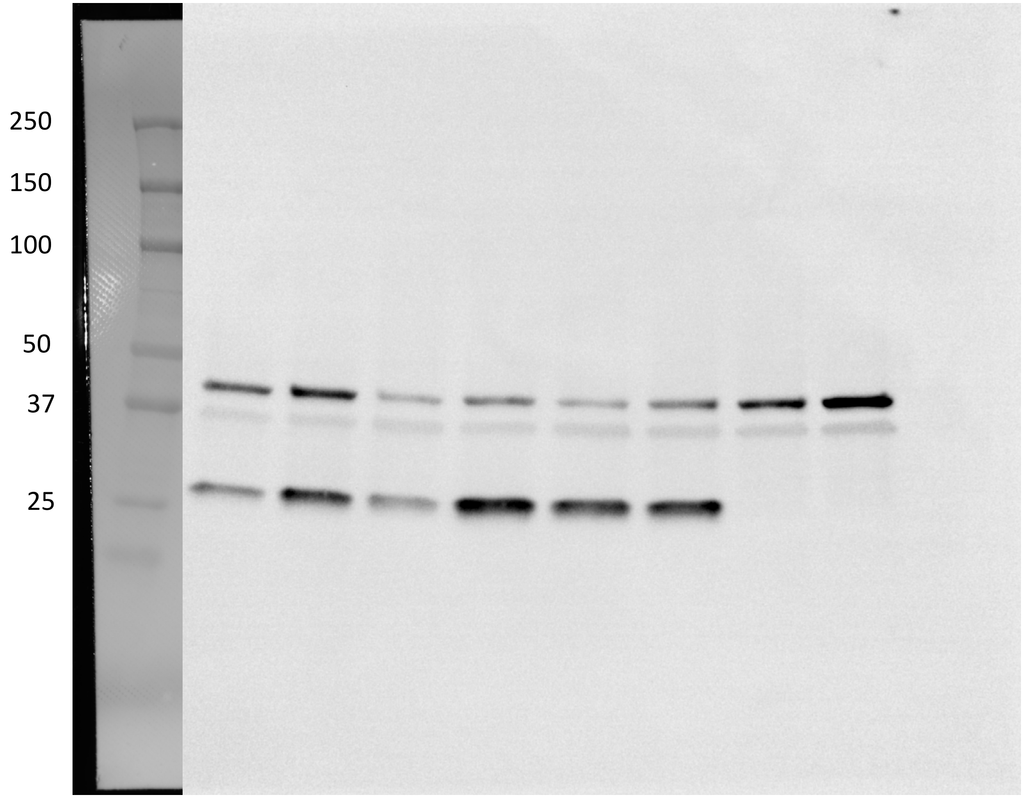

Blot 1
GAPDH 1:125,000
Mouse-HRP 1:2500

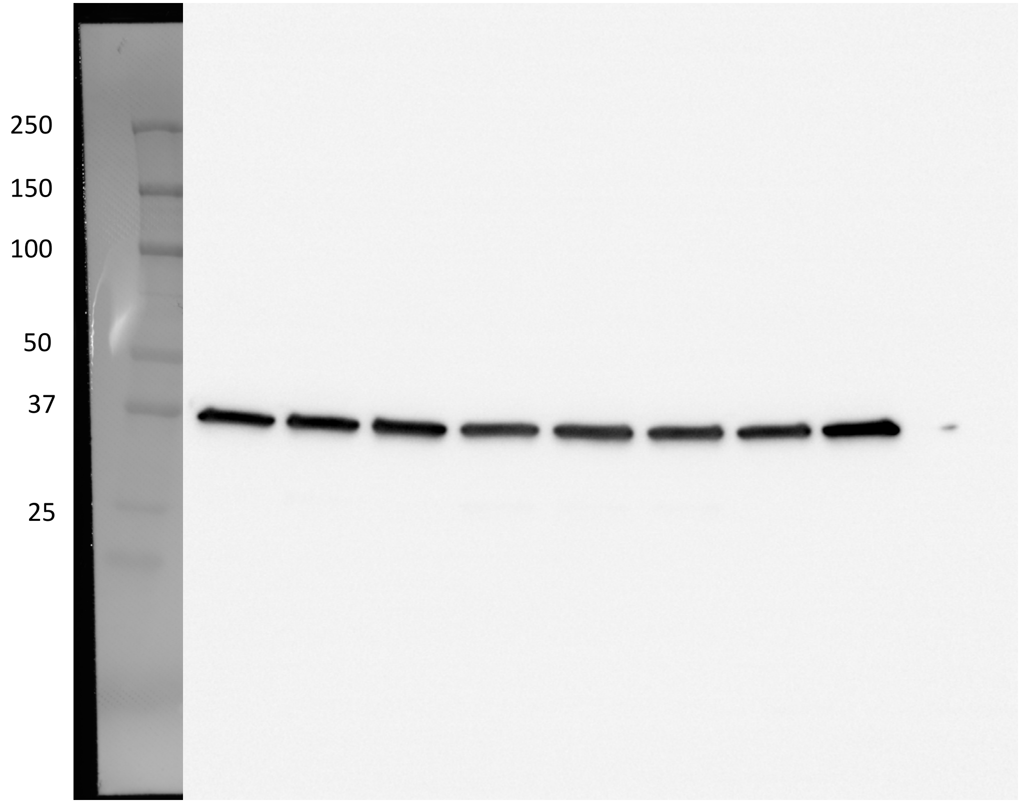

Blot 4
FBN2 1:750
Rabbit-HRP 1:2000

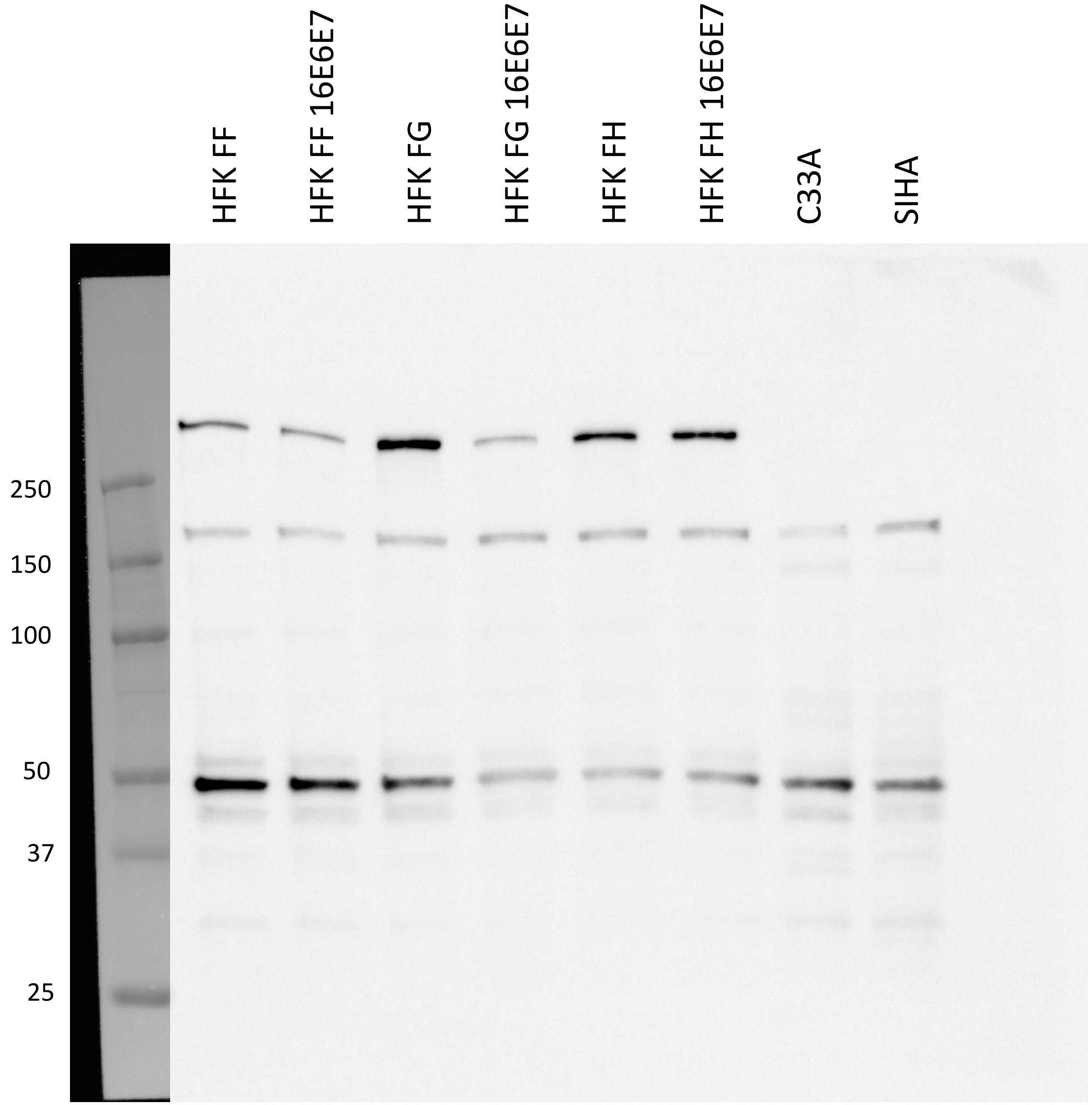

Blot 4
NFX1-123 1:1000
Rabbit-HRP 1:2000

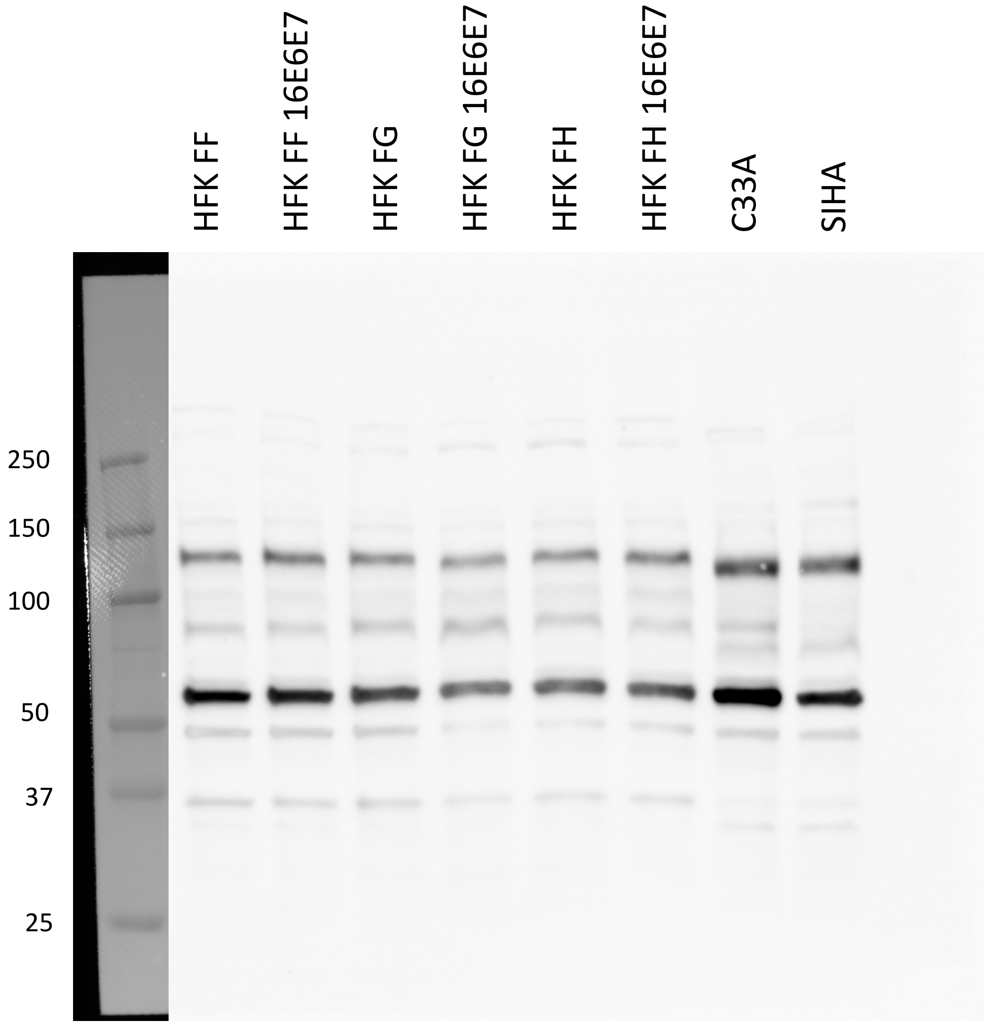

Blot 4
GAPDH 1:125,000
Mouse-HRP 1:2500

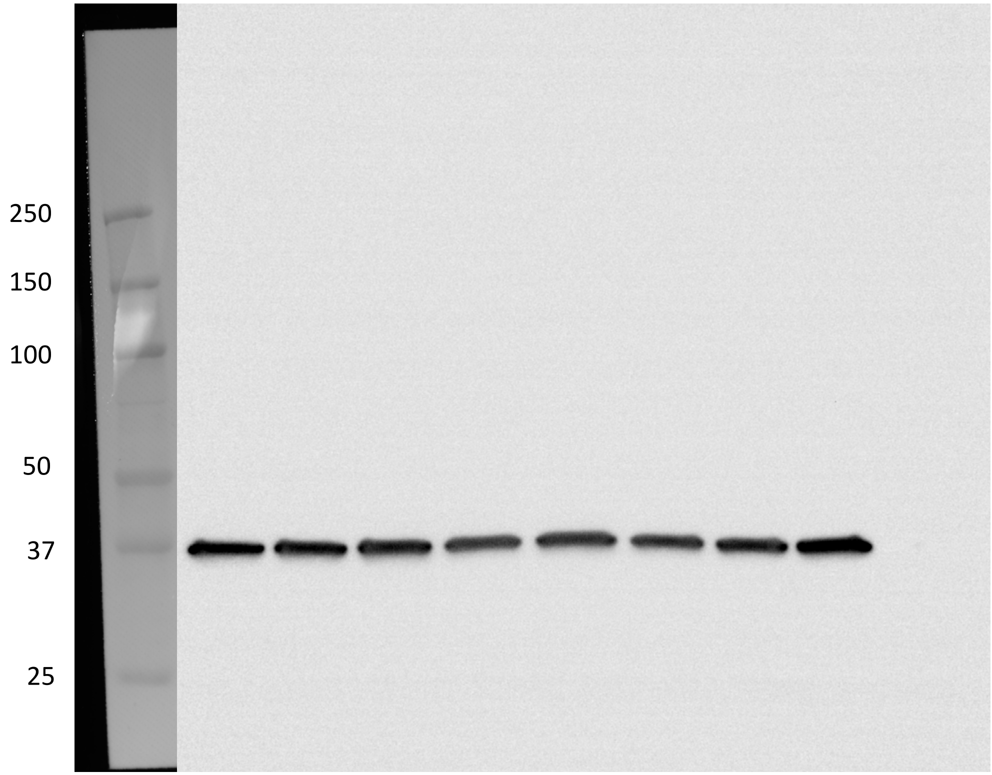

Blot 2
Rb 1:1500 Mouse-
HRP 1:2500

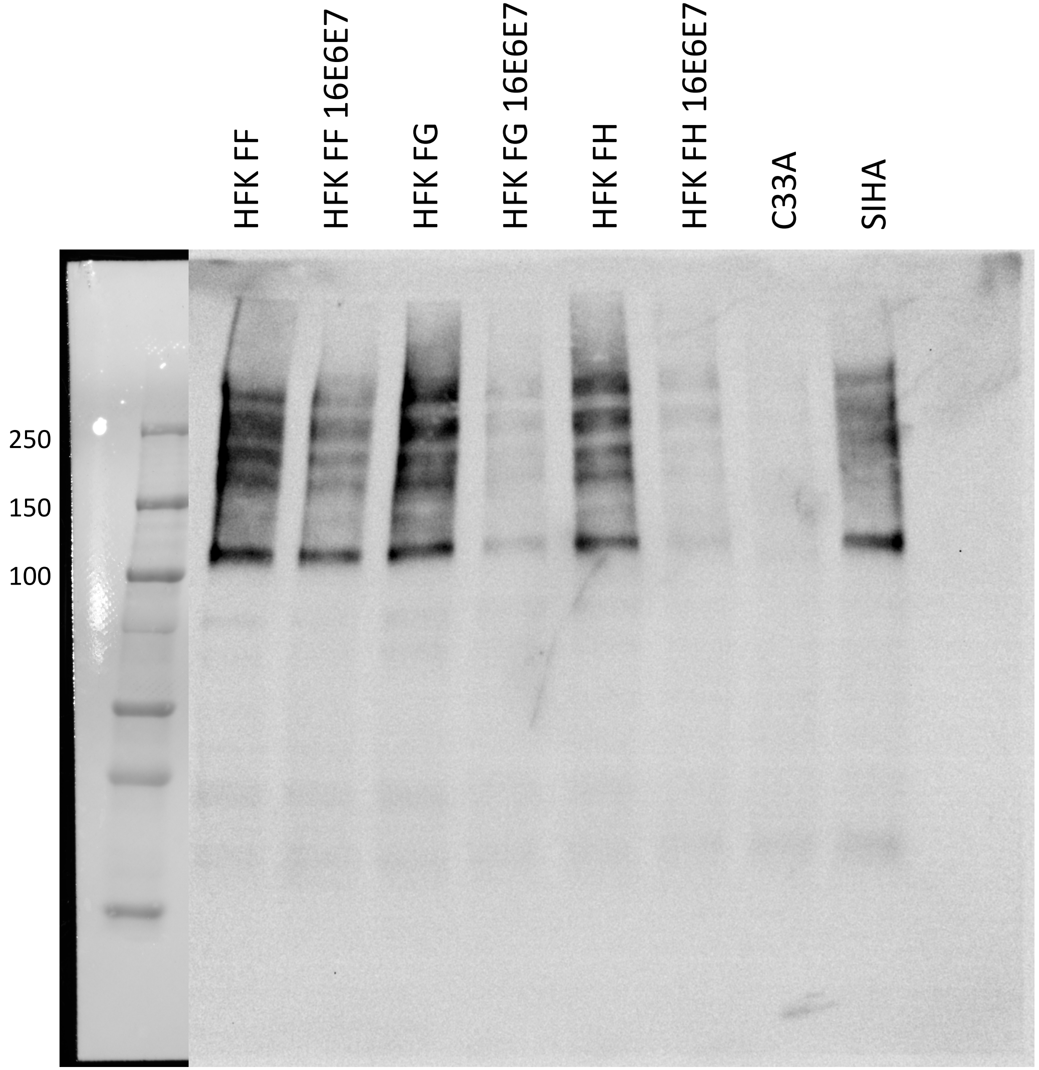

Blot 2
p53 1:1500
Mouse-HRP 1:2500

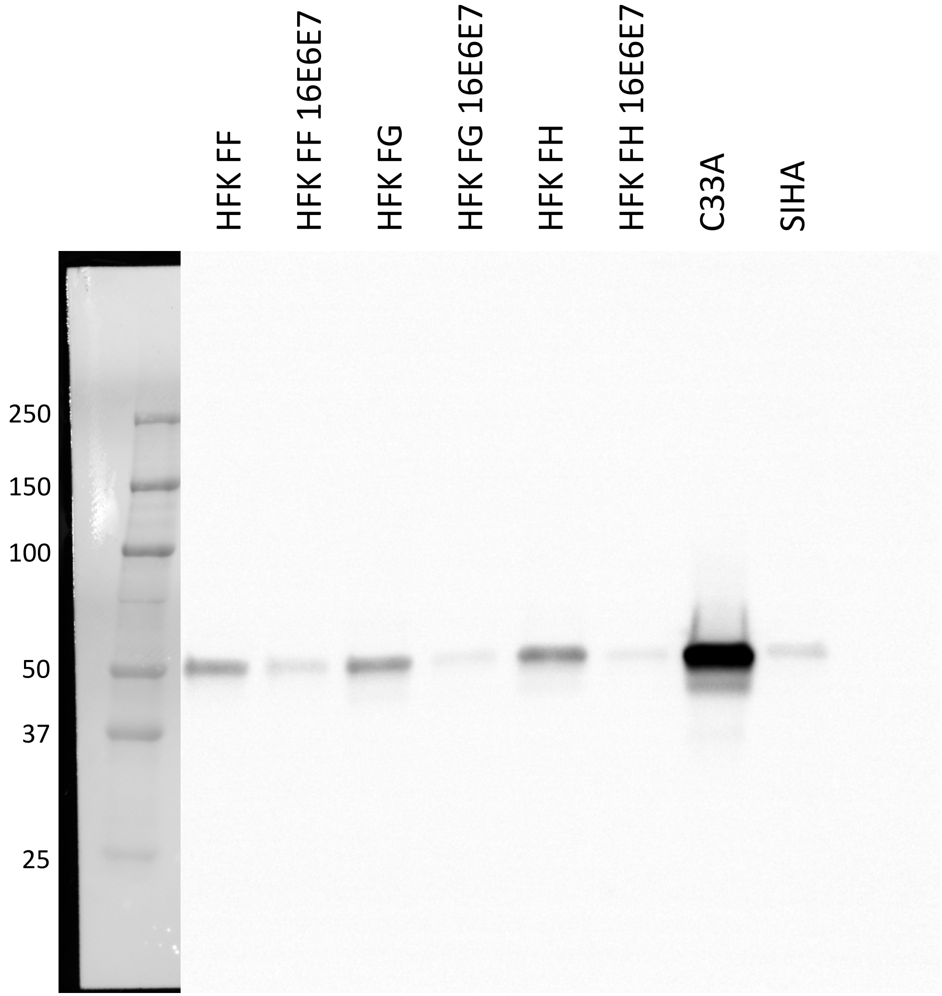

Blot 2
GAPDH 1:125,000
Mouse-HRP 1:2500

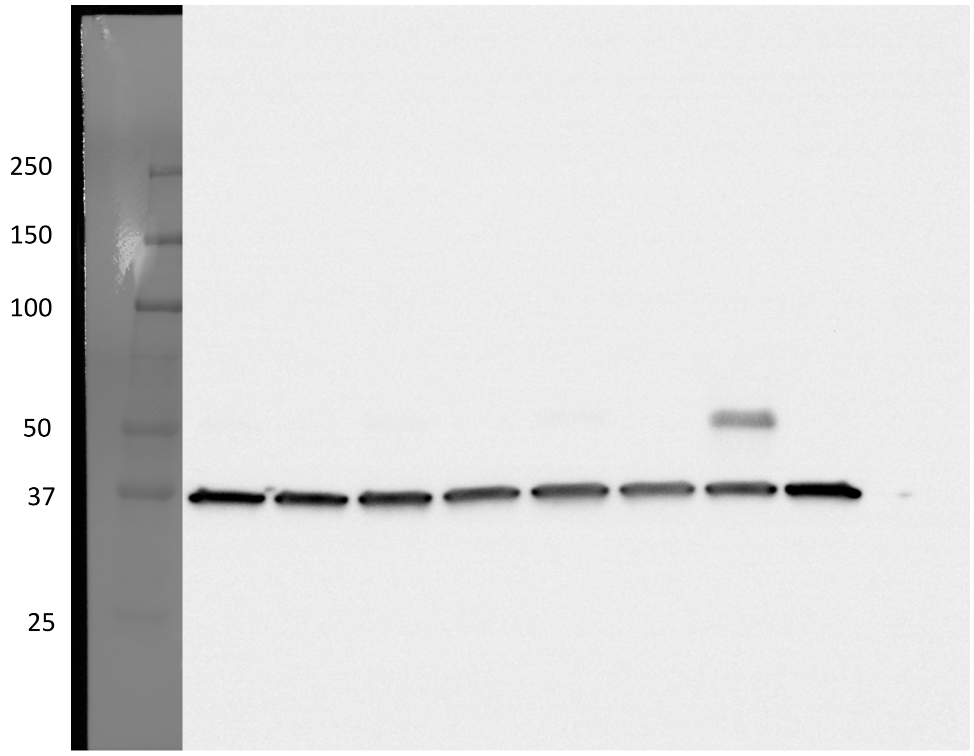

Blot 3 Notch1
1:1000 Rat-HRP
1:2000

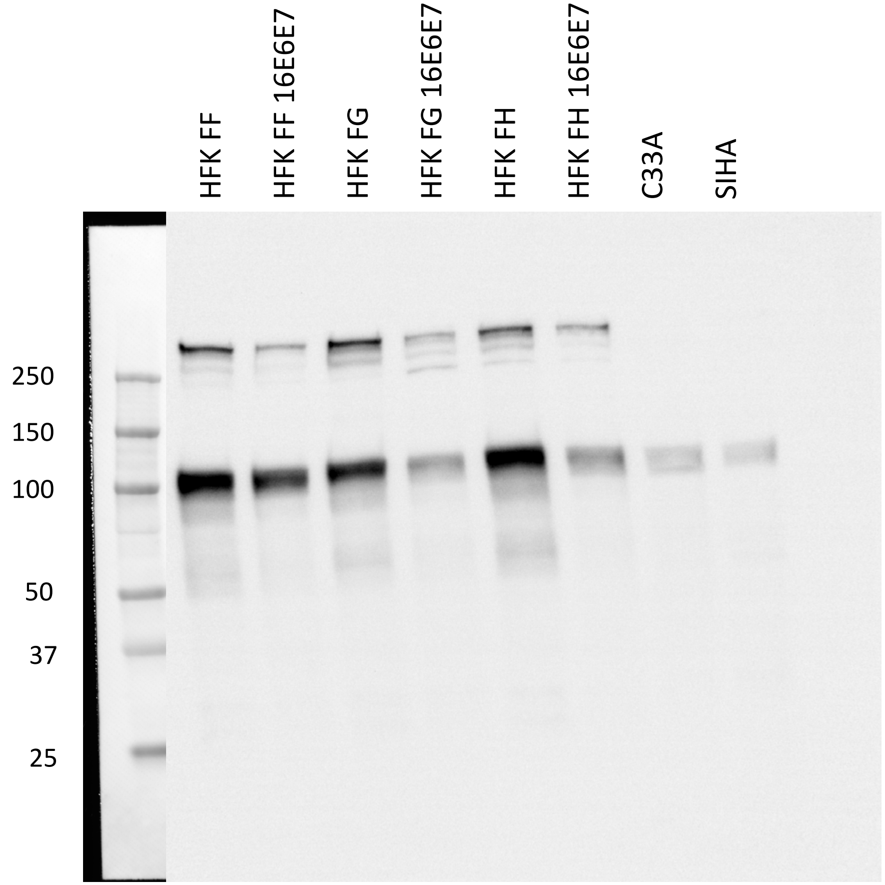

Blot 3
GAPDH 1:125,000
Mouse-HRP 1:2500

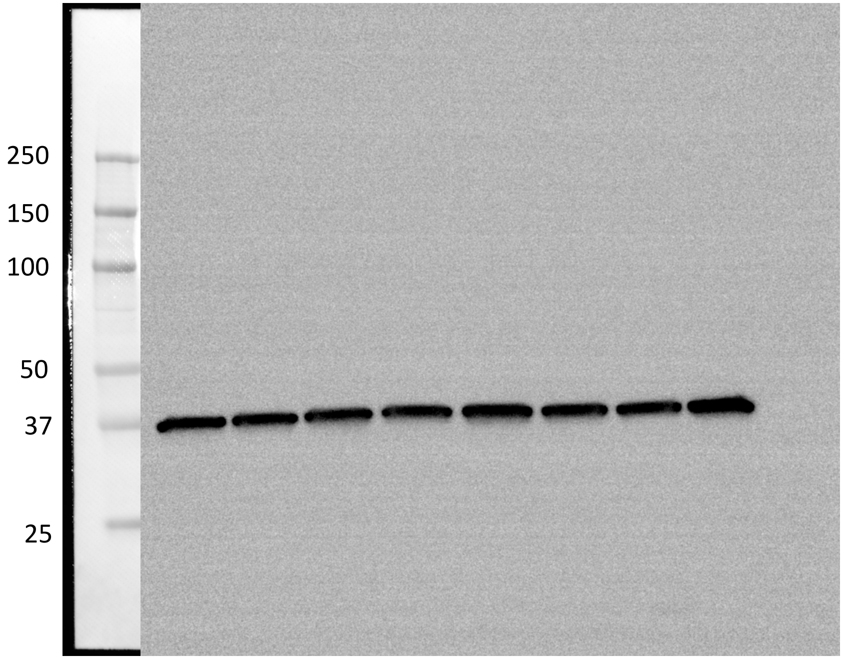

RPS29 1:2500
Rabbit-HRP 1:2000

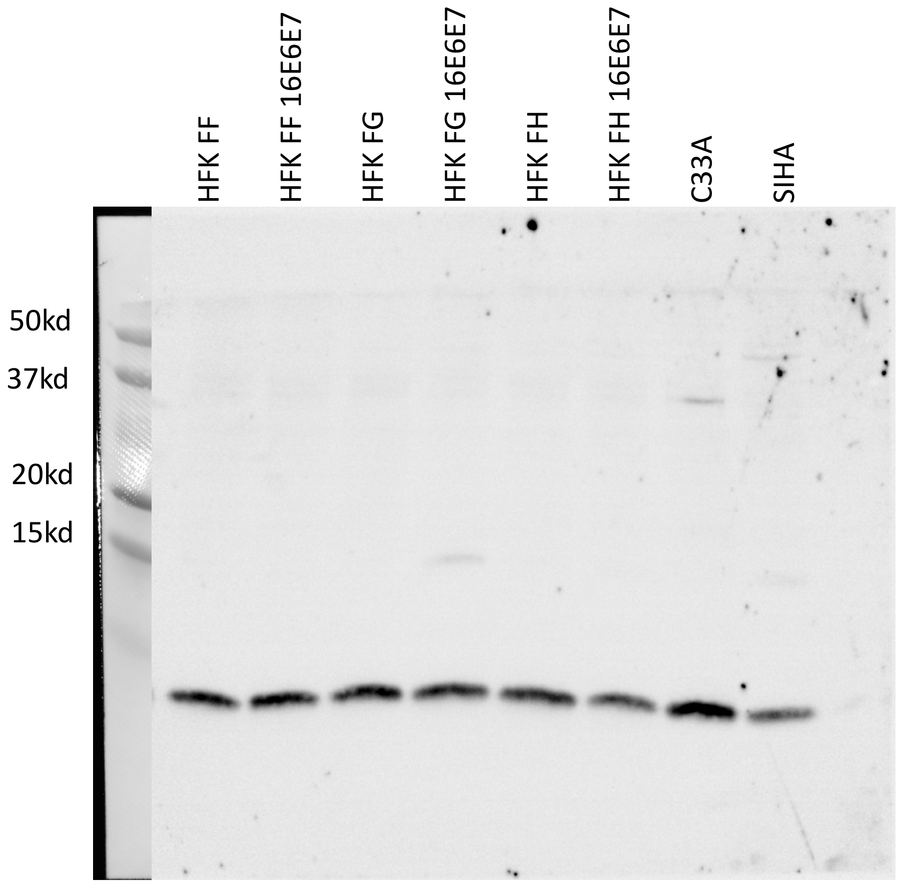

GAPDH 1:125,000
Mouse-HRP 1:2500

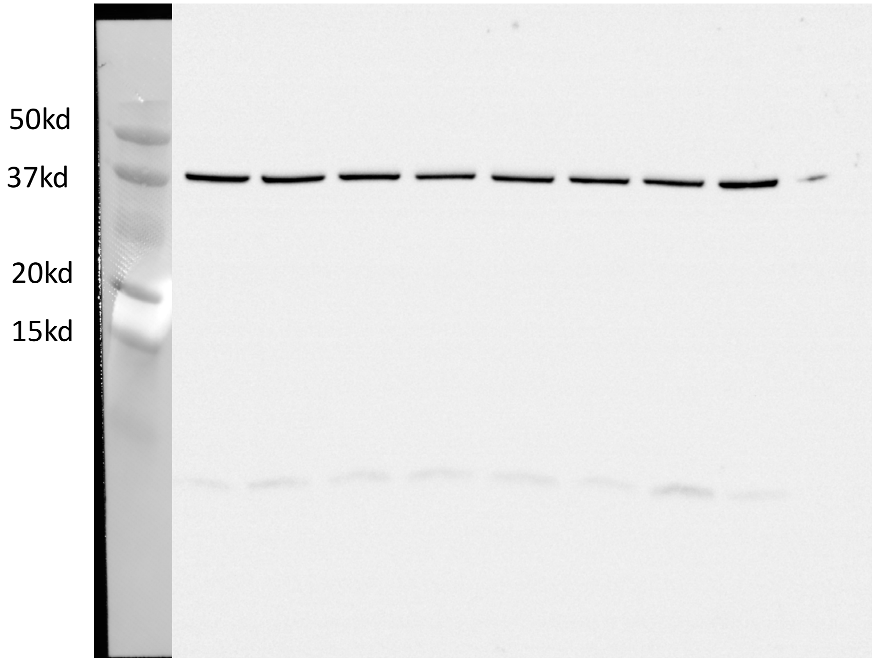

Supplement: Supplementary file 1 [file cancers-13-06182-s001.zip › Supplemental Data S1-Blots.pdf]
