# Supplementary material for: Cervical Cancer Development: Implications of HPV16 E6E7-NFX1-123 Regulated Genes
Source: Cancers (Basel). 2021 Dec 8;13(24):6182. doi: 10.3390/cancers13246182 (PMC8699269; doi:10.3390/cancers13246182)
Supplement: Supplementary file 1 [file cancers-13-06182-s001.zip › Supplemental Table S1.pdf]

Supplemental Table S1 – qPCR Primer and Probe Information and Immunoblot Antibody Information

qPCR

Primers

- KRT16
  - F – TCGAGGACCTGAGGAACAAG
  - R – GGGCCAGTTCATGCTCATAC
- NFX1-123
  - F – CCACAGCTTCCCTCCCA
  - R – CCTGGACGTCAAAATAGTCAA
- NOTCH1
  - F – GCCGAACCAATACAACCCTCTGC
  - R – GGTAGCTCATCATCTGGGACAGG
- 36B4
  - F – TGCCAGTGTCTGTCTGCAGA
  - R – ACAAAGGCAGATGGATCAGC

Probes

- CEBPD
  - HS00270931_s1
- FBN2
  - Hs00266592_m1
- GAPDH
  - Hs02786624_g1
- LCE1B
  - HS00866755_s1
- PPL
  - Hs01011417_m1
- RAB7B
  - Hs01088520_m1
- RPS29
  - Hs03004310_g1
- SLPI
  - Hs00268204_m1
- SPRR2G
  - HS00972901_s1

Immunoblot

Antibody	Company/Cat. #	Primary Dilution	Secondary	Secondary Dilution
RAB7B	Novus Biologicals – H00338382-B01P	1:1200	Mouse-HRP	1:2500
NOTCH1	Cell Signaling – #3447	1:1000	Rat-HRP	1:2000
SLPI	R&D Systems – AF1274	1:500	Goat-HRP	1:2000
RPS29	Abcam – ab56224	1:2500	Rabbit-HRP	1:2000
FBN2	ProteinTech – 20252-1-AP	1:750	Rabbit-HRP	1:2000
NFX1-123	Homemade. Kind gift of Ann Roman.	1:1200	Rabbit-HRP	1:2000
FLAG	Millipore-Sigma – F3165	1:1000	Mouse-HRP	1:2000
GAPDH	Abcam – ab8245	1:125,000	Mouse-HRP	1:2500
p53	Santa Cruz – sc-126	1:1500	Mouse-HRP	1:2500
Rb	Cell Signaling – #9309	1:1500	Mouse-HRP	1:2500

All blots and antibodies incubated in 4% non-fat dry milk in TBS-T
